# Membrane Tension Acts Through PLD2 and mTORC2 to Limit Actin Network Assembly During Neutrophil Migration

**DOI:** 10.1371/journal.pbio.1002474

**Published:** 2016-06-09

**Authors:** Alba Diz-Muñoz, Kevin Thurley, Sana Chintamen, Steven J. Altschuler, Lani F. Wu, Daniel A. Fletcher, Orion D. Weiner

**Affiliations:** 1 Cardiovascular Research Institute and Department of Biochemistry and Biophysics, University of California San Francisco, San Francisco, California, United States of America; 2 Bioengineering Department and Biophysics Program, University of California Berkeley, Berkeley, California, United States of America; 3 Dept. of Pharmaceutical Chemistry, University of California San Francisco, San Francisco, California, United States of America; Institute of Science and Technology, AUSTRIA

## Abstract

For efficient polarity and migration, cells need to regulate the magnitude and spatial distribution of actin assembly. This process is coordinated by reciprocal interactions between the actin cytoskeleton and mechanical forces. Actin polymerization-based protrusion increases tension in the plasma membrane, which in turn acts as a long-range inhibitor of actin assembly. These interactions form a negative feedback circuit that limits the magnitude of membrane tension in neutrophils and prevents expansion of the existing front and the formation of secondary fronts. It has been suggested that the plasma membrane directly inhibits actin assembly by serving as a physical barrier that opposes protrusion. Here we show that efficient control of actin polymerization-based protrusion requires an additional mechanosensory feedback cascade that indirectly links membrane tension with actin assembly. Specifically, elevated membrane tension acts through phospholipase D2 (PLD2) and the mammalian target of rapamycin complex 2 (mTORC2) to limit actin nucleation. In the absence of this pathway, neutrophils exhibit larger leading edges, higher membrane tension, and profoundly defective chemotaxis. Mathematical modeling suggests roles for both the direct (mechanical) and indirect (biochemical via PLD2 and mTORC2) feedback loops in organizing cell polarity and motility—the indirect loop is better suited to enable competition between fronts, whereas the direct loop helps spatially organize actin nucleation for efficient leading edge formation and cell movement. This circuit is essential for polarity, motility, and the control of membrane tension.

## Abstract 2

A mechanosensory biochemical cascade involving phospholipase D2 and mTORC2 coordinates physical forces and cytoskeletal rearrangements to allow efficient polarization and migration of neutrophils.

## Author Summary

How cells regulate the size and number of their protrusions for efficient polarity and motility is a fundamental question in cell biology. We recently found that immune cells known as neutrophils use physical forces to regulate this process. Actin polymerization-based protrusion stretches the plasma membrane, and this increased membrane tension acts as a long-range inhibitor of actin-based protrusions elsewhere in the cell. Here we investigate how membrane tension limits protrusion. We demonstrate that the magnitude of actin network assembly in neutrophils is determined by a mechanosensory biochemical cascade that converts increases in membrane tension into decreases in protrusion. Specifically, we show that increasing plasma membrane tension acts through a pathway containing the phospholipase D2 (PLD2) and the mammalian target of rapamycin complex 2 (mTORC2) to limit actin network assembly. Without this negative feedback pathway, neutrophils exhibit larger leading edges, higher membrane tension, and profoundly defective chemotaxis. Mathematical modeling indicates that this feedback circuit is a favorable topology to enable competition between protrusions during neutrophil polarization. Our work shows how biochemical signals, physical forces, and the cytoskeleton can collaborate to generate large-scale cellular organization.

## Introduction

Cells use multiple mechanisms of spatial signal propagation to orchestrate behaviors like polarity and motility. Well-characterized modes of information propagation include post-translational modifications, nucleotide binding and hydrolysis, and diffusion of signals from one subcellular location to another. More recently, it has become clear that physical forces also play an important role in transmitting and integrating signals in cells and tissues, where they regulate behaviors like differentiation, death, movement, and shape (reviewed in [1,2]). The most well-characterized mode of force transmission is through the cytoskeleton. However, plasma membrane tension also influences diverse cell behaviors ranging from vesicle trafficking to actin assembly [3–6].

Although the magnitude of membrane tension can differ significantly between cell types, tension is tightly controlled within individual cells (reviewed in [7]). When cells experience a change in membrane tension, they revert back to their original membrane tension setpoint within tens of minutes [8,9]. These data suggest that membrane tension is part of a self-regulating system in which cytoskeletal assembly, cell adhesion, and/or membrane trafficking adjust to keep the magnitude of membrane tension constant [3–6,8–10]. However, the mechanistic details of this circuit and how these effectors are coordinated remain unknown.

Actin polymerization-based protrusion is one of the major determinants of membrane tension in motile cells [8,11]. We recently found that membrane tension, in turn, plays a crucial role in regulating actin assembly and cell polarity during neutrophil chemotaxis. Membrane tension acts as a global inhibitor that enables sites of actin assembly to compete with one another [5]. These reciprocal interactions between biochemical signals and physical forces (protrusion increases membrane tension, which decreases protrusion) form a negative feedback circuit that limits the magnitude of membrane tension in neutrophils and prevents expansion of the existing front and formation of secondary fronts. Interfering with this negative feedback circuit impairs cell polarity and motility. For example, increasing membrane tension acts as a long-range inhibitor of leading edge signaling pathways, whereas decreasing membrane tension results in uniform activation of actin assembly [5].

How do motile cells convert increases in membrane tension to decreases in actin assembly? One important mode of regulation is thought to be a direct physical interaction in which the plasma membrane serves as a barrier that opposes protrusion. At the leading edge, where actin density is high, the resistance per actin filament due to membrane tension must be sufficiently small to allow filaments to elongate and generate protrusion. As actin density gradually decreases towards the cell sides, the membrane force per filament increases until polymerization is stalled, resulting in regions that neither protrude nor retract [11–13]. In support of this idea, a front-to-side actin density gradient has been observed in keratocytes [11], and a model consisting entirely of mechanical interactions between the actin cytoskeleton, myosin, and the plasma membrane is sufficient to predict the polarized morphologies of keratocytes [14], as well as the relation between cell shape and speed [11]. But what regulates the magnitude of actin polymerization, and, thus, membrane tension, for efficient motility? And is the purely physical role of the plasma membrane sufficient to prevent the expansion of the existing front and the formation of secondary fronts?

Here we use neutrophils to demonstrate that the magnitude of actin network assembly in chemotactic cells is determined by a mechanosensory biochemical cascade that converts increases in membrane tension into decreases in actin nucleation. Specifically, we demonstrate that increasing plasma membrane tension acts through a pathway containing the phospholipase D2 (PLD2) and the mammalian target of rapamycin complex 2 (mTORC2) to limit actin network assembly. Without this negative feedback pathway, neutrophils exhibit larger leading edges, higher membrane tension, and profoundly defective chemotaxis. Modeling suggests roles for both the direct (mechanical) and indirect (biochemical via PLD2–mTORC2) feedback loops in organizing cell polarity and motility: the direct loop promotes the formation of stable, organized zones of actin nucleation, while the indirect loop facilitates competition among emerging protrusions.

## Results

### An Increase in Membrane Tension Leads to the Activation of mTORC2

We investigated the hypothesis that an increase in membrane tension inhibits actin network assembly indirectly through a mechanosensory biochemical pathway. To search for possible regulators of this process, we prioritized signaling pathways that are modulated downstream of membrane stretch and also play a role in chemotaxis and cell polarity. Candidates such as stretch-activated calcium channels open downstream of membrane stretch in neutrophils, but calcium transients are dispensable for neutrophil polarity [15].

One attractive candidate that could link membrane stretch to inhibition of actin assembly in neutrophils is mTORC2. This complex is activated downstream of stretch in budding yeast [16] as well as epithelial and vascular smooth mammalian muscle cells [17,18]. Furthermore, mTORC2 knockdown in neutrophils (through a small hairpin RNA [shRNA] of its essential component Rictor) leads to defects in chemotaxis and more uniform accumulation of actin [19], which would be consistent with disruption of an inhibitor of actin network assembly. TORC2 plays a directed role in chemotaxis from neutrophils [19,20] to fibroblasts [21] and is essential for both chemotaxis [22,23] and electrotaxis [24] in *Dictyostelium*, suggesting that this complex may be a general regulator of directed movement.

**Fig 1:**
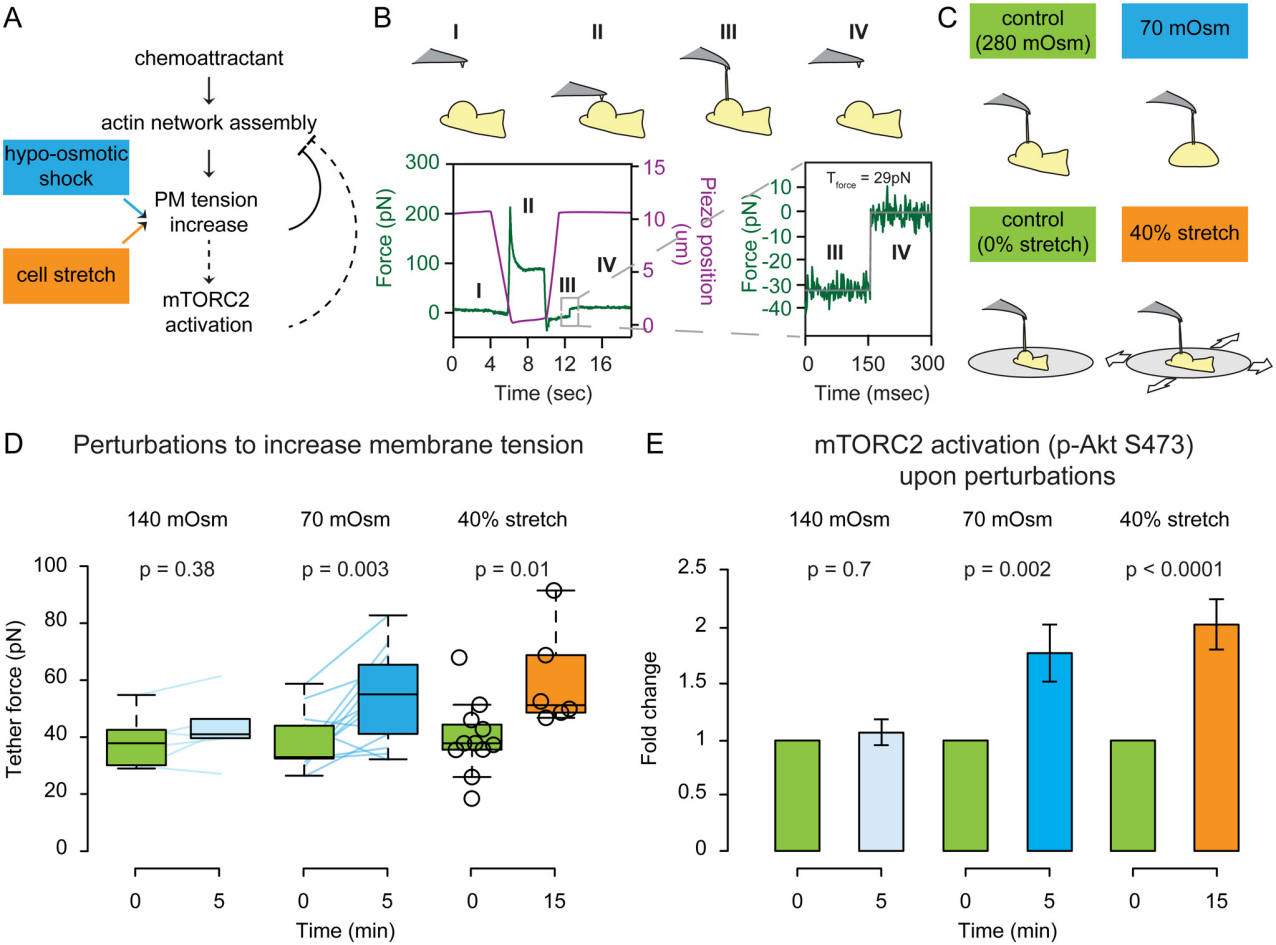
Acute stretching of the plasma membrane leads to an increase in membrane tension and an activation of mTORC2. **(A)** Existing model of direct inhibition of actin network assembly by membrane tension (solid links) with potential mTORC2-based mechanosensory pathway that converts increases in tension to decreases in actin assembly (dashed links). **(B)** Top: Schematic of tether pulling with an atomic force microscope (AFM) to measure tether force and membrane tension. Bottom: Example force–time trace showing membrane tether force quantification. The AFM cantilever (purple line indicates position of the cantilever) is brought into contact with the cell for 5 s (II) and then withdrawn (III). At this time, a membrane tether connecting the cell to the cantilever produces a negative force reading (green line). After tether breakage (IV), the force experienced by the cantilever returns to zero. The difference between the pre-breakage and post-breakage force indicates the tether force. Data fitted with the Kerssemakers algorithm (in grey). See Materials and Methods for details. **(C)** We used two perturbations to increase membrane tension. Top: Schematic of tether pulling before and after hypo-osmotic shock. Bottom: Schematic of tether pulling for cells plated on a stretchable substrate. **(D)** Static tether force for chemoattractant-stimulated cells before and after 140 or 70 mOsm hypo-osmotic shocks or 40% radial stretch. Connected colored lines indicate the same cell before and after hypo-osmotic shock. 70 mOsm hypo-osmotic shock and 40% radial stretch both increase membrane tension to a similar degree (*p* ≤ 0.01). **(E)** Left: Median of the pAkt S473 immunofluorescence peak by phospho-flow following 140 or 70 mOsm hypo-osmotic shock. Right: Mean of the pAkt S473 immunofluorescence intensity by epifluorescence before and after 40% stretch (all normalized to corresponding control untreated cells in green). Both perturbations that increase membrane tension also increase mTORC2 activation (*p* < 0.01). N_biological replicates_: D = 3 (140mOsm shock), 12 (70 mOsm shock), 2 (40% stretch). E = 5 (140mOsm shock), 7 (70mOsm shock), and 2 (40% stretch). N_cells_: D = 6 (140mOsm shock), 13 (70 mOsm shock), 11 (before 40% stretch), 6 (after 40% stretch). E >10000 (140 or 70 mOsm shock), 26 (before 40% stretch), and 33 (after 40% stretch). N_tethers_: D = 15 (before 140 mOsm shock), 13 (after 140 mOsm shock), 34 (before 70 mOsm shock), 25 (after 70 mOsm shock), 21 (before 40% stretch), 19 (after 40% stretch). Statistics: Mann-Whitney test. Boxes in all box plots (D) extend from the 25th to 75th percentiles, with a line at the median. Whiskers extend to ×1.5 IQR (interquartile range) or the max/min data points if they fall within ×1.5 IQR.

mTORC2 is a protein complex comprising the mTOR kinase, the rapamycin-insensitive companion of mTOR (Rictor), and four other proteins [25]. mTORC2 phosphorylates the serine/threonine protein kinase Akt at S473 in mammalian cells in response to a broad range of stimuli [26,27], including neutrophils stimulated with the bacteria-derived chemoattractant peptide formyl-Met-Leu-Phe (fMLP) [19,20]. Several possible inputs and outputs for mTORC2 have been identified [23,28–34], but how mTORC2 is activated downstream of chemoattractant to regulate cell motility is not well understood. We sought to investigate whether the increase in membrane tension that normally occurs downstream of chemoattractant addition [5,35,36] is sufficient to activate mTORC2 in the absence of chemoattractant stimulation (Fig 1A).

To address the effects of an increase in membrane tension on neutrophil signaling, we used neutrophil-like differentiated HL-60 cells, which recapitulate the chemotactic responses of primary blood neutrophils. We first established an experimental framework for manipulating and measuring membrane tension in a manner independent of chemotactic stimulation (Fig 1B–1D). Hypo-osmotic shocks have been extensively used to increase membrane tension by stretching the plasma membrane through osmotically-driven water influx [3,4]. To measure the magnitude of membrane tension changes, we used an atomic force microscope (AFM) to quantify the force needed to extrude a single membrane nanotube (or tether) from the plasma membrane [1,2,37,38]. Resulting force–time curves were fitted with the Kerssemakers algorithm [39] to obtain the tether force. We then estimate the magnitude of membrane tension using the formula [3–6,37]

$$T=\frac{F_{0}^{2}}{8B\pi^{2}}$$

with *F*_*0*_ being the tether force measured by AFM and *B* being the bending rigidity of the membrane, which we assume is to be invariant between the different experimental conditions tested (= 2.7 10^−19^ Nm [7,37]).

Reducing the osmolarity from 280 mOsm to 140 mOsm by adding distilled water fails to alter the tether force and does not result in a loss of polarity (Fig 1D and S1 Movie). The fact that cells can resist this amount of osmotic pressure without changing cell shape or membrane tension is interesting and suggests the existence of a compensatory mechanism. Nevertheless, the capacity of this mechanism is exceeded following larger changes in osmolarity that lead to loss of polarity. Specifically, further decreasing the osmolarity to 70 mOsm leads to an increase in tether force (Fig 1D) and a loss of polarity (S1 Movie) and results in the activation of mTORC2 (Fig 1E). To rule out indirect effects of osmotic shock on cell polarity and mTORC2 activation, we developed a cell-stretching device to increase membrane tension in a manner independent of osmotic changes. We plated cells on a fibronectin-coated polydimethylsiloxane (PDMS) substrate and performed 40% radial stretch (Fig 1C). To measure the tether force on stretched cells, we designed a cell stretcher that is compatible with AFM-based tether pulling. Membrane tension was elevated in stretched cells compared to cells plated on a pre-stretched membrane, which we used to control for any mechanical effects of the cell substrate (Fig 1D). Similar to hypo-osmotically treated cells, stretched cells also increase phosphorylation of Akt at S473, indicating mTORC2 activation (Fig 1E).

In summary, mechanical stretch of the plasma membrane induced either by hypo-osmotic shock or cell stretching leads to a rapid loss of neutrophil polarity, an increase in membrane tension, and activation of mTORC2.

### Knockdown of mTORC2 Produces a Strong Migration Defect, Larger Leading Edges, and Higher Membrane Tension

**Fig 2:**
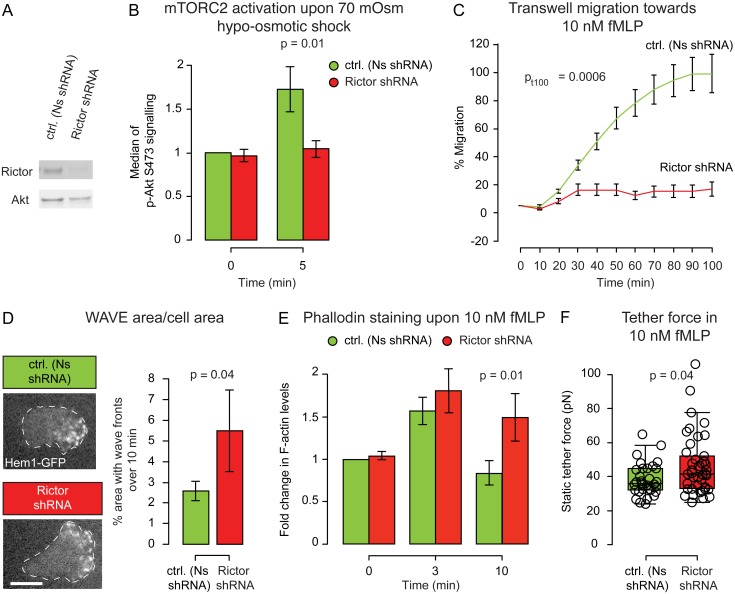
mTORC2 is an inhibitor of actin network assembly and is necessary for cell polarity, migration, and membrane tension regulation. **(A)** Representative western blot for Rictor in control and knockdown cell lines (see S2A Fig for complete blot). **(B)** Median of the pAkt S473 immunofluorescence peak (readout of mTORC2 activity) before and after a 70 mOsm hypo-osmotic shock (normalized to Ns shRNA untreated cells). Mean ± SEM. Rictor is required for the membrane stretch-induced phosphorylation of mTORC2 effectors (*p* < 0.05). **(C)** Transwell assay for chemotaxis. Mean ± SEM. Rictor knockdown cells are highly defective in chemotaxis (*p* < 0.01). **(D)** Left: Representative images of Hem1-GFP (a subunit of the neutrophil WAVE2 complex) recruitment in Nonsense and Rictor shRNA cells. Dashed white line corresponds to cell outline. Right: Quantification of area covered by wave fronts over a 10 min period. Mean ± SEM. Rictor knockdown cells have significantly larger regions of WAVE2 complex recruitment (*p* < 0.05). See Materials and Methods for how the WAVE2 area measurements were performed. See S2 and S3 Movies. **(E)** Median of the phalloidin staining before and after 3 or 10 min of fMLP stimulation. Rictor knockdown cells have more sustained actin assembly than control cells (*p* < 0.05). Mean ± SEM. **(F)** Static tether force for stimulated Nonsense and Rictor shRNA cells. Rictor knockdown cells have significantly increased membrane tension (*p* < 0.05). N_biological replicates_: B = 5. C = 7. D = 4. E = 5. F = 14. N_cells_: B >10,000/data point. C = 300,000/well. D = 11 (Ns shRNA) and 12 (Rictor shRNA). E >10,000/data point. F = 32 (Ns shRNA), 41 (Rictor shRNA). N_tethers_: F = 60 (Ns shRNA) and 83 (Rictor shRNA). Statistics: Mann-Whitney test (B, D, E, F) and *t* test (C). Boxes in all box plots (F) extend from the 25th to 75th percentiles, with a line at the median. Whiskers extend to ×1.5 IQR (interquartile range) or the max/min data points if they fall within ×1.5 IQR.

If mTORC2 is participating in a negative feedback loop to constrain actin network assembly, mTORC2 should not only be activated downstream of membrane stretch, but it should also play a role in inhibiting actin network assembly. To interfere with mTORC2 activity, we used a lentivirus-based shRNA system to stably knock down Rictor, an essential component of mTORC2 (Figs 2A and S1A, mean = 34% of the Rictor protein amount in knockdown compared to control [Ns shRNA] cells, *p*-value = 0.004).

Consistent with earlier reports, Rictor knockdown (Rictor shRNA) markedly reduced S473-phosphorylation of Akt upon chemoattractant stimulation (S1C Fig; see also [19,20]). Furthermore, Rictor shRNA also blocks the increase in pAkt S473 following 70 mOsm shock, confirming that hypo-osmotic shock leads to an increase in pAkt S473 in an mTORC2-dependent manner (Fig 2B). To corroborate the previously reported role of mTORC2 in neutrophil chemotaxis [19,20] we performed transwell chemotaxis assays. Rictor shRNA cells exhibited severe chemotactic defects (Figs 2C and S1D). This migration defect was not a consequence of defective differentiation, as the neutrophil differentiation marker CD11b was unperturbed by Rictor depletion (S1F Fig).

We previously observed that neutrophils have consolidated propagating zones of SCAR/WAVE2 complex recruitment that closely correspond to zones of membrane protrusion [40]; these focused zones of actin nucleation or wave fronts are likely important to efficiently push the membrane forward in a manner that would be difficult for fragmented nucleation. To investigate the origin of the migration defect observed in Rictor shRNA cells, we analyzed the dynamics of actin nucleation in adherent HL-60 cells by using total internal reflection fluorescence (TIRF) to visualize the neutrophil WAVE2 complex component Hem1. Rictor-depleted cells exhibited larger leading edges, as measured by the percentage area of their basal membrane covered by Hem1-GFP (Fig 2D, S2 and S3 Movies).

Since neutrophils do not require adhesion to a substrate to polarize, we further analyzed the involvement of mTORC2 in adhesion-independent regulation of actin polymerization by quantifying the overall levels of polymerized actin (F-actin) via phalloidin staining of cells that were both stimulated and fixed in suspension. Control (Nonsense, Ns shRNA) cells have an initial burst of actin polymerization upon stimulation, but once the cell is polarized, the amount of F-actin is similar to that of the resting state. In contrast, Rictor shRNA cells exhibited a significantly higher amount of F-actin even 10 min post-stimulation, suggesting a defect in leading edge restriction (Fig 2E). These data suggest that mTORC2 inhibits actin network assembly in a manner that is independent of cell adhesion.

To validate our experiments in suspension (Fig 2B and 2E), we next probed whether membrane tension changes as a function of adhesion strength. By diluting fluorescent fibronectin with a protein blocker, we titrated and measured the surface density of this adhesion molecule (S2A Fig). As expected, the density of fluorescent fibronectin is strongly correlated with the number of adhering cells (S2B Fig) and their migration speed (higher adhesion slows tail retraction and cell advance, providing a 50% decrease in migration speed over the adhesion densities tested [S2C Fig]), so we can be sure we are titrating fibronectin over a sensitive window for the cells. However, we found no change in measure membrane tension across this 10-fold range of fibronectin density (S2C Fig). These data indicate that cell adhesion is not a dominating input to membrane tension in neutrophils.

If the loss of mTORC2 breaks the negative feedback circuit that normally limits actin network assembly and, thus, limits membrane tension during neutrophil chemotaxis, we would expect elevated membrane tension in chemoattractant-stimulated Rictor-depleted cells. Consistent with this hypothesis, the average tether force for control versus mTORC2-depleted cells increased from 38 to 47 pN (Fig 2F). This corresponds to an increase in membrane tension from 69 to 103 μN/m (see above and Section II of S1 Text for details).

Taken together, these data indicate that the mTORC2 complex is activated by membrane stretch, acts as an inhibitor of actin network assembly, and is part of a pathway that establishes the magnitude of membrane tension in chemoattractant-stimulated cells.

### PLD2 Is Essential for Tension-Induced mTORC2 Activation

In mammalian cells, mTORC2 phosphorylates S473 of Akt in response to receptor tyrosine kinase stimulation, but the signaling pathway that links mTORC2 activation with upstream inputs is not well understood. In *Saccharomyces cerevisiae*, cell stretch is thought to unfold membrane invaginations known as eisosomes, releasing the TORC2 activator Slm1 [16]. Mammalian cells lack eisosomes and an obvious Slm1 orthologue, but we hypothesized that an analogous mTORC2 activator could be released from membrane invaginations in neutrophils. We searched Biogrid (Biological General Repository for Interaction Datasets, http://thebiogrid.org/) for mTORC2-interacting proteins that are also components of clathrin-coated pits or caveolae and identified PLD2 as a particularly attractive candidate. PLD2 is a phospholipase that converts phosphatidylcholine into choline and phosphatidic acid, which is an activator of the mTor kinase for both mTORC1 and mTORC2 [41,42].

**Fig 3:**
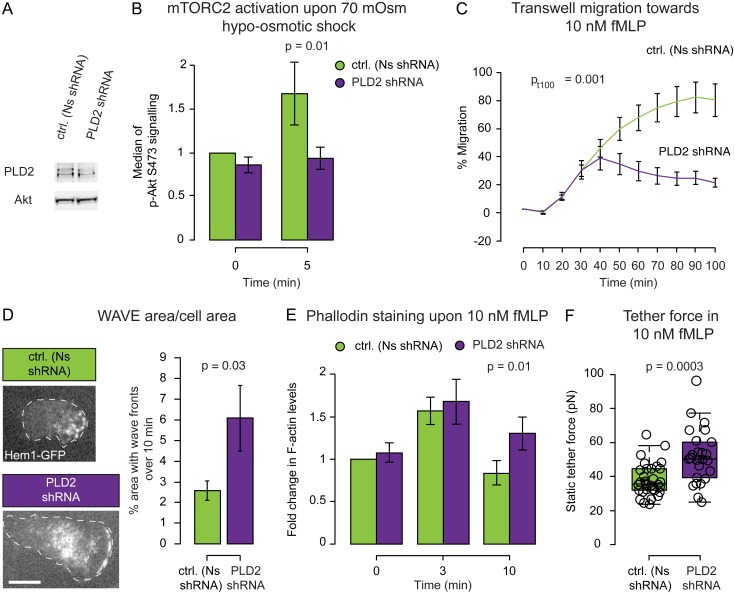
PLD2 couples membrane stretch to mTORC2 activation and supports proper actin dynamics, directed cell migration, and membrane tension regulation. **(A)** Representative western blot for PLD2 and band densitometry (see S1B Fig for complete blot). **(B)** Median of the immunofluorescence peak of pAkt 473 (as readout for mTORC2 activity) before and after a 70 mOsm hypo-osmotic shock (normalized to Nonsense shRNA untreated cells). Mean ± SEM. PLD2 is required for the membrane stretch-induced activation of mTORC2 (*p* < 0.05). **(C)** Transwell assay for chemotaxis. Mean ± SEM. PLD2 knockdown cells are highly defective in chemotaxis (*p* < 0.01). **(D)** Left: Representative images of Hem1-GFP (a subunit of the neutrophil WAVE2 complex) recruitment in Nonsense and PLD2 shRNA cells. Dashed white line corresponds to cell outline. Right: Quantification of area covered by wave fronts over a 10 min period. Mean ± SEM. PLD2 knockdown cells have significantly larger regions of WAVE2 complex recruitment (*p* < 0.05). See Materials and Methods for how the WAVE2 area measurements were performed. See S2 and S4 Movies. **(E)** Median of phalloidin staining before and 3 and 10 min after fMLP stimulation. PLD2 knockdown cells have more sustained actin assembly than control cells (*p* < 0.05). Mean ± SEM. **(F)** Static tether force for stimulated Nonsense and PLD2 shRNA cells. PLD2 knockdown cells have significantly increased membrane tension (*p* < 0.01). N_biological replicates_: B = 4. C = 8. D = 5. E = 5. F = 13. N_cells_: B >10,000/data point. C = 300,000/well. D = 11 (Ns shRNA) and 15 (PLD2 shRNA). E >10,000/data point. F = 32 (Ns shRNA), 25 (PLD2 shRNA). N_tethers_: F = 60 (Ns shRNA) and 52 (PLD2 shRNA). Statistics: Mann-Whitney test (D, E, F) and *t* test (B, C). Boxes in all box plots (F) extend from the 25th to 75th percentiles, with a line at the median. Whiskers extend to ×1.5 IQR (interquartile range) or the max/min data points if they fall within ×1.5 IQR.

To test the functional relevance of PLD2 in the regulation of mTORC2 activity and actin assembly downstream of membrane stretch, we stably expressed a PLD2 shRNA and verified the knockdown by western blot (Figs 3A and S1B, mean = 39% of the PLD2 protein amount in knockdown compared to control [Ns shRNA] cells). PLD2 knockdown cells showed a reduction in S473-phosphorylation of Akt upon chemoattractant stimulation (S1C Fig) and following a 70 mOsm shock (Fig 3B). These results suggest that PLD2 is necessary for mTORC2 signaling downstream of both chemoattractant and hypo-osmotic shock.

Whether PLD2 has a general role in cell migration is a matter of debate [43–45]. To assess the role for PLD2 in HL-60 chemotaxis, we performed transwell assays on PLD2 shRNA cells and observed a severe chemotactic defect (Figs 3C and S1E). This migration defect is not a consequence of impaired differentiation, as the CD11b differentiation marker is unperturbed by PLD2 knock down (S1F Fig).

To characterize the regulation of actin assembly in PLD2 shRNA cells, we performed TIRF imaging of the WAVE2 complex subunit Hem1 in adherent cells and quantified the amount of F-actin by phalloidin staining of cells that were both stimulated and fixed in suspension. PLD2-depleted cells exhibited both larger wave fronts and a higher amount of polymerized actin following stimulation (Fig 3D and 3E, S2 and S4 Movies). Similar to cells that were knocked down for mTORC2, PLD2 knockdown cells also have higher membrane tension upon stimulation (Fig 3F), with an increase in static tether force from 38 to 52 pN, which corresponds with an increase in membrane tension from 69 to 126 μN/m (see above and Section II of S1 Text for details). As a secondary means of reducing PLD2 activity, we used the pharmacological PLD2 inhibitor VU0285655-1 [46], which produced a similar increase in static tether force from 37 (in the dimethyl sulfoxide [DMSO]-treated control) to 53 pN, which corresponds with an increase in membrane tension from 64 to 132 μN/m (S3A Fig).

Taken together, our observations suggest that PLD2 links membrane tension increases to mTORC2 activation and that this feedback circuit helps to establish the size of the leading edge and the magnitude of membrane tension in chemoattractant-stimulated neutrophils.

### PLD2 and mTORC2 Convert Increases in Membrane Tension to Decreases in Actin Nucleation

**Fig 4:**
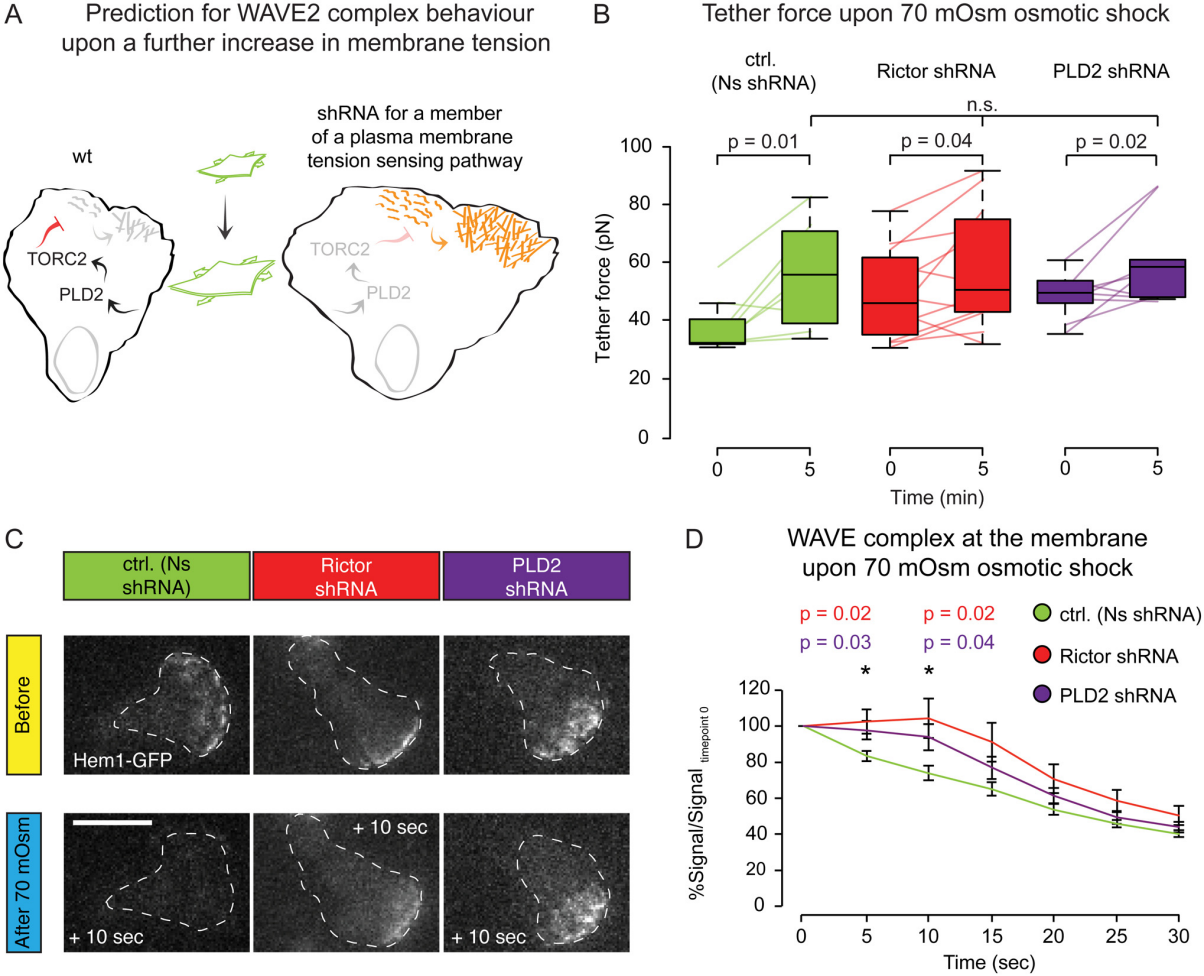
PLD2 and mTORC2 are required to convert increases in membrane tension to decreases in actin network assembly. **(A)** Prediction: Knockdown of the PLD2–mTORC2-based tension circuit should result in a slower decrease of actin nucleation following an increase in membrane tension. **(B)** Paired static tether force measurements for stimulated cells before and after 5 min of 70 mOsm hypo-osmotic shock. Connected colored lines indicate the same cell before and after hypo-osmotic shock. All conditions significantly increase their membrane tension following hypo-osmotic shock (*p* < 0.05). **(C)** Representative images of Hem1-GFP before and 10 sec after a 70 mOsm hypo-osmotic shock. Dashed white line corresponds to cell outline. Scale bar = 10 μm. Rictor and PLD2 knockdown cells are delayed in the detachment of the WAVE2 complex from the membrane following hypo-osmotic shock. See S4 Fig for complete time series of WAVE2 complex loss following 70 mOsm hypo-osmotic shock. **(D)** Quantification of the dynamics of Hem1-GFP on the membrane after 70 mOsm hypo-osmotic shock. Mean ± SEM. Rictor and PLD2 knockdown cells are delayed in the detachment of the WAVE2 complex from the membrane following hypo-osmotic shock (*p* < 0.05). N_cells_: B = 8 (Ns shRNA), 12 (Rictor shRNA), and 7 (PLD2 shRNA). D = 22 (Ns shRNA), 17 (Rictor shRNA), and 12 (PLD2 shRNA). N_tethers_: B = 17 (Ns shRNA), 19 (Rictor shRNA), and 9 (PLD2 shRNA). Statistics: paired *t* test (B) and *t* test (D). Boxes in all box plots (B) extend from the 25th to 75th percentiles, with a line at the median. Whiskers extend to ×1.5 IQR (interquartile range) or the max/min data points if they fall within ×1.5 IQR.

In control cells, increasing membrane tension inhibits actin assembly [5]. In the absence of PLD2 or mTORC2, neutrophils show enhanced actin nucleation and a more abundant actin network (Figs 2D, 2E, 3D and 3E), even though their membrane tension is significantly higher than chemoattractant-stimulated control cells ever normally achieve (Figs 2F and 3F). These data suggest that the link between membrane tension and actin assembly may be impaired in the absence of PLD2 and mTORC2. To investigate this possibility, we sought to further increase membrane tension through hypo-osmotic shock to evaluate whether the PLD2 and mTORC2 knockdown cells are defective at converting increases in membrane tension to decreases in actin network assembly (Figs 4 and S4).

We measured membrane tension in individual cells before and after osmotic shock and observed that 70 mOsm hypo-osmotic shock led to a similar elevated level of membrane tension in both control as well as PLD2 and Rictor knockdown cells (Fig 4B). Intriguingly, Nonsense, PLD2, and Rictor shRNA cells achieved the same high level of membrane tension regardless of their initial values of actin assembly or membrane tension. These data suggest that osmotic pressure dominates membrane tension in such circumstances and that a PLD2/mTORC2 independent mechanism sets an upper bound of membrane tension under these conditions.

Cells knocked down for PLD2 or Rictor were defective in stretch-induced inhibition of actin nucleation, as assessed via the loss of the WAVE2 complex component Hem1 from the membrane (Fig 4C, 4D and S4 Fig).

These data indicate that PLD2 and mTORC2 are required to efficiently relay increases in membrane tension to decreases in actin nucleation in neutrophils.

### Mathematical Modeling Suggests Complementary Roles for Direct and Indirect-Based Feedback to Actin Assembly

Previous studies suggest that membrane tension reduces actin polymerization directly by providing a barrier to its growth [11]. Our experimental data provide evidence that a separate inhibitory link from PLD2–mTORC2 to the WAVE2 complex is necessary for limiting actin assembly and controlling membrane tension. Here we investigated how these negative feedback links to actin assembly—direct (mechanical via the membrane as a physical barrier) and indirect (biochemical via PLD2–mTORC2 mechanosensory cascade)—might collaborate to regulate cell polarity and motility.

Since not all involved cellular processes are understood in sufficient detail for a complete mechanistic description, we sought to develop a conceptual model that allowed us to understand the contribution of the two different feedbacks. We used a previously developed mathematical model for simulating the propagating spatial dynamics of actin and the Hem-1 component of the WAVE2 complex in a small portion of the membrane in neutrophils [40]. In this previous model, stochastic WAVE2 complex activation—represented by WAVE2 binding to the membrane—is enhanced by self-association. Recruited WAVE2 complex promotes the nucleation of actin filaments, and actin polymerization inhibits further binding of the WAVE2 complex on the membrane via local negative feedback [47].

**Fig 5:**
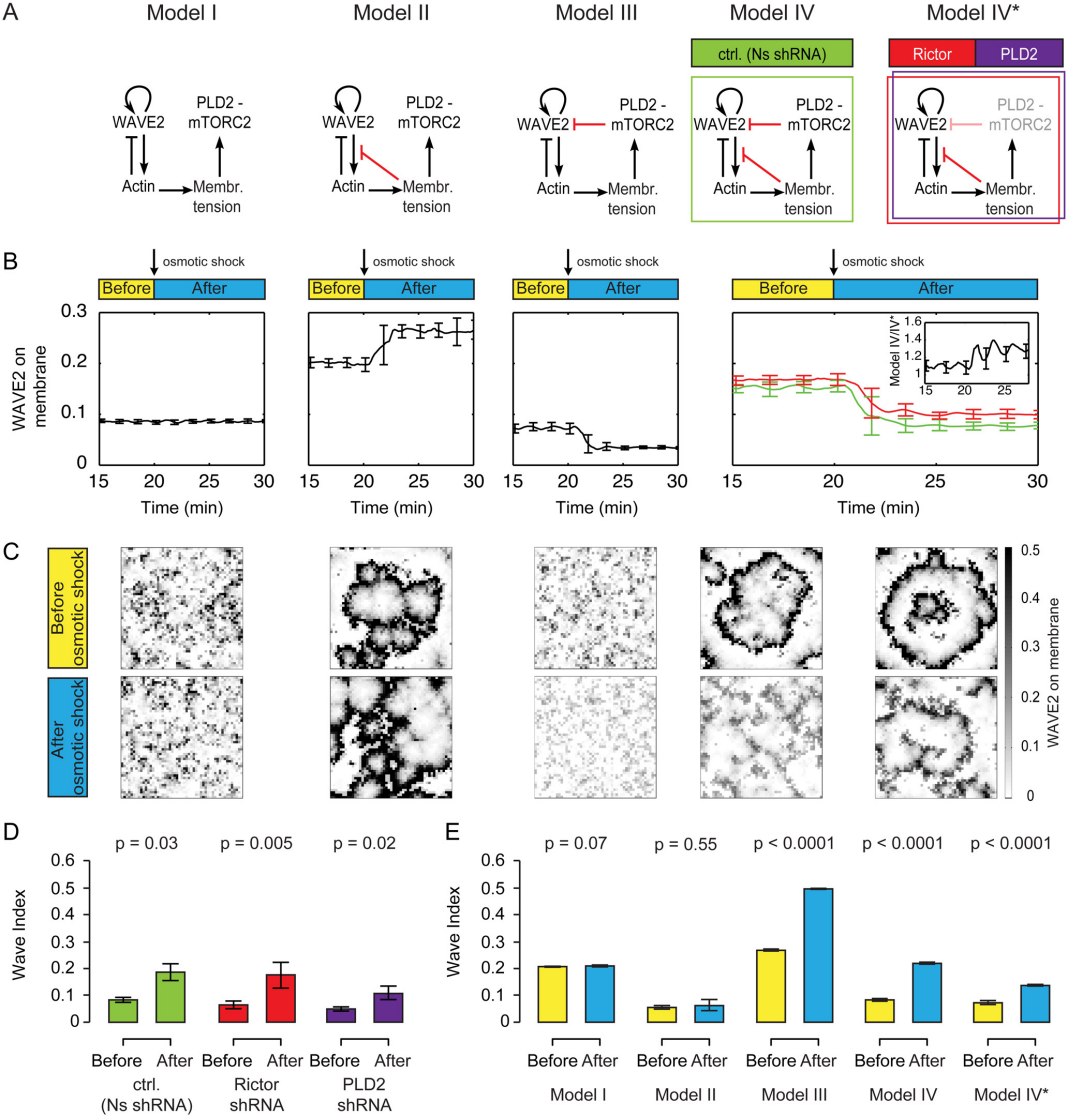
Probing possible topologies of membrane tension-based inhibition of actin network assembly. **(A**) Schematic of the different network topologies investigated. Model I: WAVE2 complex dynamics in the absence of tension-mediated inhibition of actin polymerization (see also S5B Fig for early time points). Model II: Global inhibition of actin polymerization by membrane tension only. Model III: Global inhibition of the WAVE2 complex by mTORC2 only. Model IV: Two distinct negative feedbacks from membrane tension to actin network assembly (from tension to actin polymerization and from PLD2–mTORC2 to the WAVE2 complex). Model IV*: Model IV with reduced mTORC2 mediated feedback strength, reflecting our shRNA lines. See Materials and Methods and S1 Text for details of the simulation. **(B**) WAVE2 complex on the membrane before and after hypo-osmotic shock in the different models. Mean ± SD of 20 stochastic simulations. Only the models with the link from PLD2–mTORC2 to WAVE2 (Model III and Model IV) match our experimental observation of tension-based decreases in WAVE2 association with the membrane (Fig 4C and 4D). Inset: Ratio of models IV, IV*, showing that the difference is larger after osmotic shock. **(C**) Snapshots of typical simulations before and after osmotic shock (simulated as a step increase in membrane tension of 80 μN/m). See S5 Movie. **(D**) Quantification of the Wave Index (WI) in control (Ns shRNA), Rictor, and PLD2 shRNA cells before and after osmotic shock. Mean ± SEM. Data used from Fig 4D. **(E)** Quantification of the Wave Index in the different models before and after osmotic shock. Mean ± SD of 20 stochastic simulations. Model IV exhibits the expected increase of WI upon osmotic shock and shows a similar level of WI to experimental cells (Fig 5D). N_cells/simulations_: D = 20 (Ns shRNA), 15 (Rictor shRNA), and 13 (PLD2 shRNA). E = 20 each condition. Statistics: Mann-Whitney test.

We then extended the model (Model I in Fig 5A, S5A and S5B Fig) to include feedback from membrane tension (Models II–IV in Fig 5A and S5A Fig). Specifically, we modeled networks in which feedback from membrane tension directly (Model II), indirectly (Model III), or both directly and indirectly (Model IV) inhibit actin polymerization. We additionally analyzed a model with reduced strength of indirect feedback (Model IV* in Fig 5A) to simulate PLD2 and Rictor knockdown experiments (Figs 3 and 4).

We assume that basal membrane tension (before hypo-osmotic shock) is proportional to the total amount of polymerized actin (S5C Fig, see also [8]) and the hypo-osmotic shock-based increase in membrane tension activates the PLD2–mTORC2 pathway in a switch-like manner (S5D Fig and S1 Text). We performed several lines of experiments to test these assumptions.

To verify that membrane tension is proportional to the total amount of polymerized actin, we used low doses of the monomer sequestering drug latrunculin B (50 nM) as a general inhibitor of actin polymerization and the drug CK666 inhibitor to specifically inhibit Arp2/3 complex-mediated actin assembly (100 μM). We quantified the overall levels of polymerized actin (F-actin) via phalloidin staining and showed, as previously reported [48,49], that low doses of latrunculin and CK666 partially blocked chemoattractant-induced actin polymerization (S5E Fig). Latrunculin B produced a profound decrease in the average tether force from 37 pN in control DMSO-treated cells to 11 pN (S5F Fig). This corresponds to a decrease in membrane tension from 64 to 6 μN/m (see above and Section II of S1 Text for details). CK666 produced a more moderate decrease in membrane tension (from 37 pN in control DMSO-treated cells to 23 pN; S5F Fig), corresponding to a decrease in membrane tension from 64 to 25 μN/m. These data suggest that actin polymerization in general, and the Arp2/3 complex in particular, play a significant role in generating membrane tension in neutrophils. Furthermore, for the perturbations tested, membrane tension scales linearly with the amount of actin polymerization (S5C Fig).

To verify that the PLD2-mTORC2 pathway is activated in a switch-like manner, we assayed the activation of this pathway over a range of membrane tension values (S5G Fig). In particular, we used actin polymerization inhibitors to decrease membrane tension (S5F Fig) and hypo-osmotic shock and cell stretching to increase membrane tension (Fig 1D), and we assessed mTORC2 activation by assessing S473-phosphorylation of Akt (Fig 1E). Together, these experiments show that mTORC2 activation responds nonlinearly to increases in membrane tension and suggested this relationship is well described by a Hill coefficient of ~8 (S5G and S5H Fig). We tested this result against curves with fixed, lower Hill coefficients (S5H Fig) and found that the best-fit curve explains the data significantly better than models with Hill coefficient ≤3 (*p* = 0.03, F test), thus justifying the assumption of the switch-like nature of PLD2-mTORC2 activation.

The SCAR/WAVE2 complex is a critical regulator of actin nucleation and directed migration [50–56], and its spatial and temporal dynamics closely correlate with actin assembly and morphological rearrangements in a wide range of motile cells [57–61], including neutrophils [40]. Therefore, we used the abundance and spatial patterning of WAVE2 as the main readout of the model and as the point of comparison to experimental data (Figs 2D, 3D, 4C and 4D). After a transient period, all models reach a stationary state with only minor stochastic fluctuations (S6A Fig), as they all contain the local negative feedback involving actin’s inhibition of the SCAR/WAVE2 complex. As expected, in the absence of the direct and indirect links from membrane tension to actin assembly (global feedback, Model I in Fig 5A), WAVE2 fails to respond to a simulated hypo-osmotic shock (Fig 5B, simulated by an increase in membrane tension of 80 μN/m, similar to the experiment in Fig 4). The addition of a direct link from membrane tension to actin polymerization (Model II in Fig 5A, S6B Fig) results in an increase in WAVE2 complex recruitment following an increase in tension due to the double-negative feedback via actin. This increase is not consistent with our experimental observations (Fig 4C and 4D). We found that the observed detachment of WAVE2 complex from the plasma membrane upon hypo-osmotic shock is only reproduced in the models containing the PLD2–mTORC2 inhibition of WAVE2 complex recruitment (Models III, IV, IV* in Fig 5A and S6B Fig). Furthermore, a marked WAVE2 complex detachment only occurs if the PLD2–mTORC2 pathway is activated by membrane tension in a switch-like manner (Hill coefficient ≥3, S7A Fig). As in the experiments (Fig 4D), disruption of the indirect feedback (mimicking PLD2 or mTORC2 knockdown) caused a slightly weaker response to osmotic shock (Fig 5B, 50 +/-1% decrease in Model IV versus 41 +/-1% in Model IV* after shock).

We next investigated the spatial organization of WAVE2 before and after osmotic shock (Fig 5C–5E). We quantified WAVE2 spatial patterning by a Wave Index (WI), which is a metric of disorganization calculated from the number of connected components in the spatial organization of WAVE2. A smaller WI indicates a smaller number of connected waves and a higher degree of spatial organization (see Materials and Methods). Analysis of experimental data (S4 Fig) reveals coherent waves before osmotic shock and an increase in the WI upon hypo-osmotic shock, indicating a loss of WAVE2 complex organization (Fig 5D). So an increase in tension not only decreases the amount of WAVE2 complex at the membrane (Fig 4C and 4D), but the portion that remains is also less organized (Fig 5D). These results are qualitatively unchanged for five times higher or lower values of most model parameters (ratio plots in S7B Fig).

Our base model (Model I), which lacks both links from membrane tension to actin, exhibits moderately disorganized waves (WI ≈ 0.2) that predictably do not change upon osmotic shock (Fig 5E). Addition of the direct link from tension to actin polymerization (Model II) resulted in significantly more organized waves (WI ≈ 0.1), but this did not change following osmotic shock (Fig 5E). Both models that contained the inhibitory link from mTORC2 and PLD2 to WAVE2 (Models III and IV) exhibited an increase in WI upon osmotic shock, but only the dual inhibition model (Model IV) had coherent waves before osmotic shock consistent with our experimental data (Figs 2D, 3D and 5C–5E). Furthermore, decreasing the strength of the indirect link (Model IV*) recapitulated the experimental data for PLD2 and mTORC2 knockdowns of delayed kinetics of WAVE2 disappearance following osmotic shock (Figs 4D and 5B) and attenuated increase of WI upon hypo-osmotic shock (Fig 5D and 5E). We conclude that a model that contains both the direct and indirect links from tension to actin assembly is most consistent with our experimental observations.

To analyze the relative impact of both feedbacks on WAVE2 complex spatial organization, we varied the strength of each link individually (S6B Fig). The direct restriction of actin network growth (together with the [local] actin-mediated inhibition of WAVE2) results in a positive link from membrane tension to the WAVE2 complex, resulting in a stabilization of wave fronts; this leads to a higher degree of organization of actin nucleation. In contrast, the PLD2–mTORC2 biochemical mechanosensor provides an indirect inhibitory link to the WAVE2 complex, and this couples tension increases to decreases in the WAVE2 complex on the membrane, potentially forming the basis of competition between sites of actin protrusion. We explicitly test this competition in the following simulations.

### Competing Sites of Wave Initiation Suppress One Another

**Fig 6:**
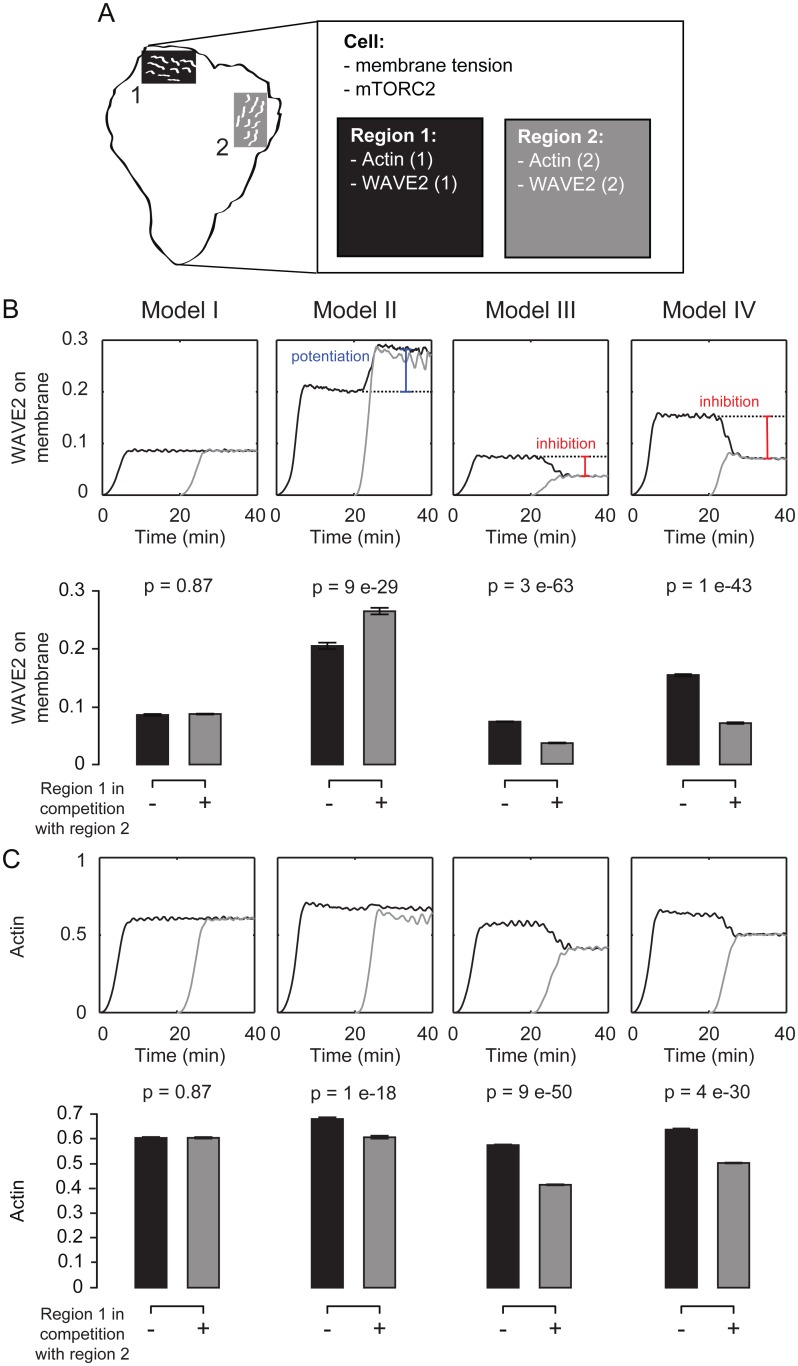
Simulated competition between two nucleating regions that share membrane tension. **(A**) Schematic of competition simulations. We simulated the response of two spatially separate sites of actin assembly that are linked via membrane tension. The two regions each have individual dynamics of actin polymerization and WAVE2 membrane binding but are coupled via cellular membrane tension and level of mTORC2 activity. **(B,C**) Quantification of time-varying (graphs) and time-averaged (bar plots) WAVE2 complex (B) and polymerized actin (C) at the membrane. A site of actin nucleation (number 1) starts alone, but is then placed in competition with a second site of actin nucleation (number 2). Mean ± SD of 20 stochastic simulations. Only Models III and IV show competition between protrusions at the level of WAVE2 complex recruitment. See S8 Fig for how competition affects the Wave Index. Statistics: *t* test.

Our simulations suggest that the PLD2–mTORC2 link is necessary for membrane tension to influence the magnitude and spatial dynamics of the WAVE2 complex. We hypothesized that this link could play an important role in enabling actin protrusions to cross-inhibit one another. To investigate this possibility, we simulated the response of two spatially separate sites of actin assembly that are linked only by membrane tension (Fig 6A). Thus, actin polymerization and WAVE2 membrane binding are computed separately for each region, and both contribute to cellular membrane tension and subsequent mTORC2 activity.

We analyzed the activity of one protrusion when it grows in isolation (Fig 6A, region 1 alone, black) or following equivalent activation of polymerization in a second region (activity of region 1 [black] followed by activation of region 2 [grey], Fig 6B and 6C, S8 Fig, S6 Movie and S1 Text). Only the models that contain the inhibitory link from PLD2 and mTORC2 to the WAVE2 complex (Models III and IV) exhibit inhibition of WAVE2 complex recruitment and a loss of spatial organization by a secondary front, as can be seen in the higher Wave Index following competition (S8 Fig). The model that contains only the direct link from membrane tension to actin polymerization (Model II) exhibits a potentiation of WAVE2 complex recruitment during competition. This compensatory increase in WAVE2 complex recruitment buffers the tension-mediated decrease in actin polymerization, resulting in a minimal change in overall actin mass for the direct link alone (Fig 6C), making Model II a poor topology for competition. In contrast, the models with the indirect PLD2–mTORC2 link (Models III and IV) are more efficient in competition between fronts, on the level of actin polymer accumulation as well as for the amount and spatial organization of the WAVE2 complex (Figs 6B, 6C and S8).

Taken together, our model suggests that membrane tension plays two complementary roles to regulate actin nucleation and polymerization for cell polarity. The direct (mechanical) restriction of actin polymerization stabilizes the spatial organization of wave fronts while the indirect inhibitory link to the WAVE2 complex (PLD2–mTORC2 based) restricts nucleation and enables competition between protrusions for efficient leading edge formation and movement.

## Discussion

### Plasma Membrane Tension Inhibits Actin Nucleation through a Mechanosensory-Based Negative Feedback Circuit

**Fig 7:**
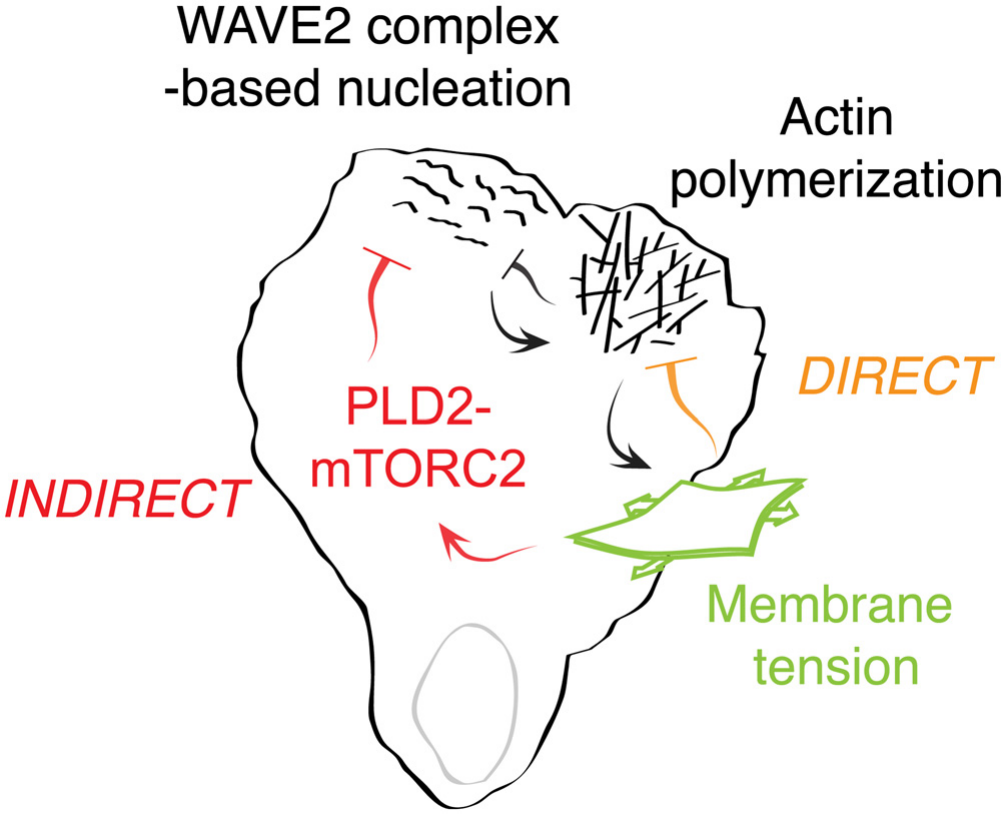
Working model of PLD2–TORC2 membrane tension negative feedback loop. Actin assembly increases membrane tension, which activates two inhibitory links to actin network growth. In the first link, membrane tension directly inhibits actin polymerization by acting as a physical barrier to growth. In the second link, membrane tension acts through the PLD2–mTORC2 pathway to inhibit actin nucleation via the WAVE2 complex. This circuit forms a mechanosensory negative feedback loop that regulates membrane tension and controls the spatial organization of actin assembly during neutrophil polarity and movement.

Membrane tension is thought to regulate leading edge dynamics by acting as a direct physical barrier to actin polymerization [11]. Similarly, when neutrophil protrusions are stalled by contacting a physical barrier, the actin nucleation machinery is inhibited [25,40]. We find that membrane tension also acts as an inhibitor of actin network assembly in an indirect manner: increases in plasma membrane tension activate the PLD2–mTORC2 pathway, and these components are essential for efficiently converting increases in membrane tension to decreases in actin nucleation. Our theoretical model suggests that these direct and indirect inhibitions collaborate to ensure the proper number, size, and spatial organization of cellular protrusions (Fig 7).

Upon disruption of the PLD2–mTORC2 pathway, neutrophils exhibit sustained high levels of F-actin in response to chemoattractant, higher membrane tension, and severely defective chemotaxis. Thus, the PLD2–mTORC2 pathway forms a mechanosensory-based negative feedback loop that limits actin nucleation, organizes cell polarity, and limits the magnitude of membrane tension. Our work demonstrates how the plasma membrane integrates physical forces and intracellular signals to organize cell polarity and membrane tension during movement. Furthermore, we place PLD2 and mTORC2 into a new mechanosensory context for eukaryotic chemotaxis.

Using a mechanosensory pathway to regulate actin network assembly could give cells more flexibility in tuning membrane tension and leading edge dynamics than if they were to only rely on direct physical coupling. Our work indicates that membrane tension can affect actin organization through the PLD2–mTORC2 signaling pathway, but, importantly, there are other known (potentially orthologous) signaling inputs into PLD2 and mTORC2 that could affect PLD2 and mTORC2 activation. Global modulation of this pathway could enable different cell types to establish different set points for membrane tension. Local modulation of this pathway could enable different thresholds of actin inhibition for different regions of a cell. Furthermore, membrane tension is lower in the presence of the PLD2–mTORC2 pathway, meaning that the protrusions that are formed have less membrane tension to push against and may be able to extend more efficiently than would be the case in the absence of PLD2 and mTORC2.

It is likely that multiple pathways regulate membrane tension in neutrophils. In response to 140mOsm hypotonic solutions, neutrophils maintain their polarized state and resting membrane tension, indicating that they can compensate for moderate changes in osmotic pressure (Fig 1D). At physiological levels of membrane tension, the mTORC2–PLD2 pathway plays an important role, as actin dynamics and membrane tension are defective in their absence. At high levels of osmotic pressure (by 70 mOsm hypotonic solutions), wild-type, mTORC2, and PLD2 KD cells all reach the same membrane tension limit (Fig 4B), suggesting a PLD2/mTORC2 independent mechanism that sets an upper bound of membrane tension under these conditions.

mTORC2 and PLD2 act downstream of membrane tension to inhibit the WAVE2 complex, but this is not the only means of converting increases in membrane tension to decreases in actin nucleation. An alternative pathway has recently been proposed to link membrane tension to actin assembly via NWASP/WASP modulation [62]. FBP17 is a BAR-containing NWASP/WASP regulator that binds membrane invaginations. Elevated tension releases this protein from the membrane, providing a potential link between increases in membrane tension and decreases in NWASP/WASP-based actin assembly [62]. Whether this pathway plays a role in neutrophil polarity, in which WAVE2 complex-dependent actin assembly dominates over NWASP/WASP-based actin assembly, is not known. Moreover, in neutrophils, the PLD2–mTORC2 axis appears to play a substantial role, as cell polarity, actin assembly, and membrane tension are all significantly defective in its absence. We suggest that multiple mechanisms of responding to changes in membrane tension could regulate different cytoskeletal programs, operate at different thresholds of tension, or dominate in different cell types.

Rictor and PLD2 shRNA cells have higher magnitude of membrane tension following chemoattractant stimulation. This is an unusual phenotype, as chemoattractant-stimulated neutrophils normally have a highly stereotyped value of membrane tension. Even perturbations that affect resting membrane tension (like inhibition of myosin II by blebbistatin) fail to alter membrane tension following chemotactic stimulation [5]. Moreover, in keratocytes, even perturbations that affect the number or size of the leading edge (like myosin phosphatase knockdown) don’t change the value of membrane tension [63].

Our data suggest that for neutrophils, membrane tension regulation operates through a negative feedback circuit involving PLD2 and mTORC2. Actin polymerization increases membrane tension, activating the PLD2–mTORC2 pathway, which in turn inhibits actin nucleation to establish an upper limit on leading edge size and the magnitude of membrane tension.

### The Role of PLD2 in Leading Edge Formation

How might PLD2 connect increases in membrane tension to the activation of the mTORC2 pathway in neutrophils? One possible clue comes from the tension-based regulation of the TORC2 pathway in budding yeast. In *S*. *cerevisiae*, membrane invaginations known as eisosomes restrict the activity of Slm1, an activator of TORC2 [16]. Increases in membrane tension liberate Slm1 from eisosomes, freeing it to stimulate TORC2. There are no eisosomes or Slm1 orthologues in mammalian cells, but membrane invaginations and PLD2 could play a functionally similar role. PLD2 interacts with components of the mTORC2 complex and membrane invaginations (clathrin coated pits and caveolae [64,65]) and could be regulated in a similar manner to Slm1. Importantly, there is evidence that membrane reservoirs play an important role in neutrophil polarity and motility [66], and both caveolae and clathrin coated pits have been shown to unfold or arrest upon an increase in membrane tension [3,4]. Future studies should focus on identifying the nature of such membrane reservoirs in mammalian cells and on how PLD2 is regulated downstream of changes in membrane tension.

### mTORC2-Based Regulation of Actin Network Assembly

We observe that knockdown of the mTORC2 subunit Rictor severely inhibits neutrophil polarization and migration following chemoattractant stimulation, and this protein complex is essential to link increases in membrane tension to decreases in WAVE2-mediated actin nucleation. Several reports point to a pivotal role for mTORC2 in regulating neutrophil polarity during chemotaxis [19,20] but it remains unclear if the primary functions of mTORC2 in neutrophil motility depend on the mTor kinase activity or reflect kinase-independent protein interactions of the complex [20].

Upon hypo-osmotic shock, we observe a detachment of WAVE2 complex from the membrane. This may represent an inhibition upstream of the WAVE2 complex, as effects of stretch on Rac activity have been previously reported in neutrophils and other systems [5,67]. Interestingly, genetic loss-of-function perturbations for TORC2 yield a range of cytoskeletal defects in different cellular systems, possibly reflecting the existence of both positive and negative inputs to actin network assembly with different relative strengths in different contexts [23,34,68–71].

### PLD2–mTORC2 in Homeostasis and Chemotaxis

Plasma membrane tension impinges on a broad range of cellular events: it determines the leading edge size in neutrophils [5], streamlines polymerization in the direction of movement in *Caenorhabditis elegans* sperm cell [72], determines the folding state of caveolae [4], orchestrates phagocytosis [73] and cell spreading [74], and regulates the balance between exocytosis and endocytosis in a wide range of cell types [3,6,10,75]. Could the PLD2–mTORC2 pathway be a relevant player in these disparate cellular events?

TORC2 signaling downstream of changes in membrane tension appears to be conserved through evolution from yeast [16] to mammalian cells [17,18]. Furthermore, PLDs are activated in muscle cells after stretch and in erythrocytes upon hypo-osmotic shock [41,76]. Moreover, PLD2 is necessary in early phases of cell spreading [77], when membrane tension also plays a role [78].

An attractive idea is that the PLD2–mTORC2 pathway is part of a homeostatic system for regulating and responding to changes in membrane tension that motile cells have co-opted to regulate polarity and motility. Future studies will focus on testing whether the PLD2–mTORC2 is an integral component of other membrane-tension regulated processes and further elucidating the molecular links from membrane tension to the polarity and motility machinery.

## Materials and Methods

### Cell Lines and Culture

HL-60 cells were grown in RPMI-1640 media with L-glutamine and 25 mM HEPES (10–041-CM, Mediatech) containing: 10% heat-inactivated fetal bovine serum (FBS; Life Technologies, 16140–071), penicillin, and streptomycin (UCSF Cell Culture Facility) at 37°C with 5% CO_2_ in a humidified incubator. Cell differentiation was initiated by adding 1.5% DMSO (endotoxin-free, hybridoma-tested; D2650, Sigma) to cells in growth media. Cells were used 4–5 d post-differentiation. Each independently-differentiated batch of HL60 cells was considered a biological replicate. For experiments requiring cell starvation, cells were starved for 1 h in RPMI-1640 media with L-glutamine and 25 mM HEPES containing 0.3% bovine serum albumin (BSA, endotoxin-free, fatty acid free; A8806, Sigma).

### Knockdown Line Generation

The shRNA sequences used in this study were:

- Control Nonsense (Ns) shRNA: 5′-CTTACTCTCGCCCAAGCGAGAG-3′
- Rictor shRNA: 5′-TTTAGTATATCTGGAATCACGT-3′
- PLD2 shRNA: 5′-GAATAAGAGGCTTGACCCTGC-3′

Nonsense and Rictor shRNA sequences were obtained from [19], and PLD2 shRNA sequence was purchased from Sigma (NM_002663.3-3195s21c1). Each shRNA was cloned into the pMK1200 lentiviral plasmid for shRNA expression [79]. This allowed puromycin selection of positive cells (0.65 μg/ml for 2 wk). Next, cells were sorted for high expressers by fluorescence-activated cell sorting on a FacsAria2 (Beckton-Dickinson), and a bulk-sorted population consisting of fluorescence positive cells was used for each experiment.

### Lentivirus Production

HEK293T cells (ATCC) were grown to 70% confluency in a 6-well plate for each lentiviral target and transfected using 1.5 μg lentivirus plasmid, 160 ng VSV-G, and 1.3 μg CMV 8.91 with TransIT-293T transfection reagent (MIR 2705, Mirus Bio) according to manufacturer’s instructions. Viral supernatants were collected 2 d after transfection, passed through a 0.45 μm filter, and concentrated using Lenti-X concentrator (631231, Clontech) according to manufacturer’s instructions. Concentrated virus was used for infection immediately or kept at -80°C for long-term storage. For lentiviral infection, 50–100 μl of each concentrated virus was added directly to 10^5^ undifferentiated HL-60 cells in the presence of 4 μg/ml polybrene. Viral media was replaced with normal growth media 24 h post-infection. Cells were sorted for high expressers by fluorescence-activated cell sorting on a FacsAria2 (Beckton-Dickinson), and a bulk-sorted population consisting of fluorescence-positive cells was used for each experiment.

### CD11b Staining

Differentiation was confirmed with PE-conjugated anti-CDllb Mouse Anti-Human mAb (clone ICRF44; A18675, Life Technologies). In brief, 2*10^5^ undifferentiated or differentiated cells were pelleted and incubated in 7 μl of anti-CD11b antibody on ice for 30 min, washed with ice-cold PBS with 1% FBS, and re-suspended in the same buffer at 10^6^ cells/ml density for analysis. PBS with 1% FBS was used to establish background signal with unstained cells.

### Total Internal Reflection Fluorescence (TIRF) Microscopy

For imaging and immunofluorescence experiments, 96-well glass bottom Microwell plates (MatriPlate by Brooks Life Science Systems) were coated for 30 min with 100 μl of 10 μg/ml fibronectin (which we prepared from whole porcine blood) and then washed with PBS. 3*10^5^ cells were plated on each dish in normal growth media and allowed to adhere for 10 min at 37°C with 100 nM formyl-Met-Leu-Phe (fMLP; F3506, Sigma).

For WAVE2 complex de-recruitment from the plasma membrane upon osmotic shock (Fig 4), cells were hypo-osmotically shocked with ddH_2_O containing 0.2% BSA and 100 nM fMLP.

TIRF images were acquired on a Nikon Ti Eclipse inverted microscope with a 60X Apo TIRF 1.49 NA objective and an electron-multiplying charge-coupled device (EM-CCD) camera (Andor iXon) controlled by NIS-Elements (Nikon, Melville, New York). Sample drift was minimized using an autofocus system (Perfect Focus; Nikon). A 488 nm laser line (200 mW) was supplied from Agilent MLC400. This laser launch uses acousto-optic tunable filters (AOTFs) to control laser output to a single-mode TIRF fiber for imaging. TIRF imaging was performed with 10 mW or less laser power, achieved through AOTF and neutral density-based laser attenuation. NIS-Elements was used for image acquisition.

### Image Processing

For WAVE2 complex de-recruitment quantification (Fig 4D), the Hem1-GFP intensity inside the cell was integrated and the background intensity of a non-cell region subtracted using a custom-made MATLAB (MathWorks, R2012a) script. For WAVE2 area measurements (Figs 2D and 3D) a “wave mask” was obtained after applying a 2-pixel Gaussian blur filter. The mask threshold was manually chosen for Nonsense shRNA cells, and the same threshold applied to all Rictor and PLD2 cells imaged on the same day (to account for variability in imaging conditions from day to day) using Fiji. For quantification, the wave mask area was integrated using a custom-made MATLAB script.

All quantifications were normalized to the cell area at each time point. To obtain an accurate “cell mask,” an additional oblique illumination angle image was obtained. The resulting time series was denoised in collaboration with John Sedat, using Priism, a software developed by Jerome Boulanger [80]. Default parameters were chosen for a two-dimensional time series, except for the patch size, which was 3 × 3. The effect of the denoising was to remove speckle noise from the background while minimizing feature loss from the cells, which enabled automatic segmentation of the cell using Fiji.

The source code is available on request to the corresponding authors.

### pAkt 473 Phospho-flow

After starvation, cells where either stimulated with 10 nM fMLP or hypo-osmotically shocked with ddH2O containing 0.3% BSA. At appropriate time points (as stated in Figs 2, 3B and S2C), cells were fixed in 4% paraformaldehyde (CytoFix; 554655 BD Biosciences) containing phosphatase inhibitors (40 mM NaF, 20 mM beta-glycerol phosphate [50020, Fluka], and PhosSTOP [4906845001, Roche]) and the protease inhibitor cOmplete, EDTA-free (11873580001, Roche). The samples were spun at 2,000 xg for 5 min to pellet and washed with DPBS. Cells were permeabilized for 30 min at -20°C in ice-cold methanol. Next, cells were washed once in DPBS and blocked in IF buffer (DPBS + 5% FBS + 2 mM EDTA) for 1 hr. Samples were washed 3 × 5 min in IF buffer and incubated for 1 h at room temperature (RT) in primary antibody diluted 1:50 anti-pAktS473 (4060S, Cell Signaling) into IF buffer. Samples were washed 3 × 5 min in IF buffer and incubated for 30 min at RT in fluorescent secondary antibody diluted 1:300 Alexa 488-Donkey-Anti-Rabbit (711-546-152, Jackson Inmuno Research) into IF buffer. After 3 × 5 min washes in IF buffer, cells were analyzed on a FACS LSRII (BD Biosciences). Data analysis and visualization was performed on FlowJo (TreeStar, Ashland, Oregon). For each sample, the median from the Alexa 488-Donkey-Anti-Rabbit Gaussian was obtained in FlowJo and normalized to the corresponding untreated control.

### pAkt S473P Immunostaining on Polydimethylsiloxane (PDMS)

Four cm^2^ pieces of PDMS were mounted on our custom-made cell stretcher, and a central region was coated with 500 μl of 10 μg/ml fibronectin (which we prepared from whole porcine blood) for 30 min, and then washed with PBS. 6*10^5^ cells were plated on stretched or unstretched PDMS (control cells were plated in pre-stretched PDMS pieces, which we used to control for any mechanical effects of the cell substrate) and allowed to adhere for 10 min at 37°C with 10 nM fMLP (F3506, Sigma). After 15 min of 0% or 40% radial stretch, cells were fixed in 4% paraformaldehyde (CytoFix; 554655 BD Biosciences) containing phosphatase inhibitors (40 mM NaF, 20 mM beta-glycerol phosphate [50020, Fluka] and PhosSTOP [4906845001, Roche]) and the protease inhibitor cOmplete, EDTA-free (11873580001, Roche). Cells were permeabilized with 0.2% triton for 10 min and washed twice with DPBS with inhibitors. Next, cells were blocked in DPBS with inhibitors and 4% BSA for 1 hr. Samples were incubated for 1 h at RT in primary antibody diluted 1:200 anti-pAktS473 (4060S, Cell Signaling) into DPBS with inhibitors. Samples were washed 3 × 5 min in DPBS and incubated for 30 min at RT in fluorescent secondary antibody diluted 1:300 Alexa 488-Donkey-Anti-Rabbit (711-546-152, Jackson Inmuno Research) into DPBS. After 3 × 5 min, PDMS pieces were covered with SlowFade Gold antifade reagent with DAPI (Life Technologies, S36938) and a glass coverslip and sealed with nail polish. Epifluorescence microscopy through the coverslip was performed on a Nikon Eclipse Ti microscope.

### F-actin Staining by Phalloidin

5*10^5^ HL-60 cells were stimulated with addition of 10 nM fMLP, fixed with 3.7% paraformaldehyde in intracellular buffer (140 mM KCl, 1 mM MgCl2, 2 mM EGTA, 320 mM sucrose, and 20 mM HEPES with pH 7.5) and incubated on ice for 15 min. After centrifugation at 2,000 xg for 5 min, the cell pellet was re-suspended in intracellular buffer containing 0.2% Triton X-100 and 1:200 Phalloidin-Alexa 488 (Molecular Probes, A12378) and stained for 15 min. Cells were then centrifuged, washed twice, and re-suspended in intracellular buffer before being analyzed on a FACS LSRII (BD Biosciences). Data analysis and visualization was performed on FlowJo (TreeStar, Ashland, Oregon) as follows: For each sample the median from the Phalloidin-Alexa 488 Gaussian was obtained in FlowJo and normalized to the corresponding untreated control.

### Drug Treatments

For latrunculin B, CK666, and VU0285655-1 experiments, cells were preincubated for 10–20 min with either solutions of 10 nM fMLP with 50 nM latrunculin B (Sigma), 100 nM fMLP with 100 μM CK666 (Sigma), or 100 nM fMLP with 4.5 μM VU0285655-1 (Avanti lipids).

### Fibronectin Surface Coating

Different adhesive substrates were obtained by coating surfaces with a range of % molar mixture of BSA and Fibronectin till saturations for over 1 h. Ten percent of such Human plasma fibronectin (Gibco) was labeled with Alexa Fluor 647 NHS-ester.

Surfaces were then washed twice with DPBS. 5*105 HL-60 cells were plated on each dish in normal growth media and allowed to adhere for 10 min at 37°C. After, cells were washed and imaged in growth media with 10 nM fMLP or membrane tension was measured in RPMI with 2% FBS. Cells were manually tracked/counted using Fiji (image processing package of ImageJ).

### Western Blot

For western blots, 106 cells were lysed in 20% ice-cold trichloroacetic acid (TCA). The samples were spun at 20,000xg for 15 minutes to pellet. The sample pellets were washed twice with 0.5% ice-cold TCA and resuspended in Laemmli protein sample buffer (161–0737, BioRad) containing 5% *β*-mercaptoethanol. Protein bands were separated by SDS-PAGE gel electrophoresis, transferred to nitrocellulose, blocked with Odyssey block, and incubated at 4°C overnight with 1:1000 dilutions of anti-Rictor (Bethyl, A300-459A) or 1:1000 anti-PLD2 (Sigma, WH0005338M1) and 1:2000 anti-Akt (40D4, Cell Signaling). The blot was developed with the fluorescent secondary antibodies Goat anti-Rabbit IRDye 680RD (Licor, 926–68071) and Goat ant-Mouse IRDye 800CW (Licor, 926–32210), and protein bands were imaged using Odyssey Infrared Imaging System (Li-COR, Biosciences).

### Transwell Assays

3*10^5^ HL-60 cells were stained with DiD (V-22887, Life Technologies) and plated on the upper chamber of a 24-well format HTSFluoBlokTM Multiwell Insert System (3 μm pore size; RF351156, BD Falcon) in RPMI without phenol red (Life Technologies, 11835–030) with 2% FBS. Cells were allowed to migrate towards the bottom well containing 10 nM fMLP for 2 hours at 37°C. The migrated cells were measured by fluorescence from the bottom of the insert, while the opaque filter prevented excitation of cells on top of the filter. Analysis was performed with a FlexStation 3 Microplate Reader (Molecular Devices). Each condition was run in triplicate, and the migration index was calculated by dividing the amount of signal in the sample well by the signal in a well in which 300,000 cells were plated in the bottom compartment.

### Membrane Tension Measurements

For membrane tension measurements, custom-made chambers were coated for 30 min with 500 μl of 20 μg/ml fibronectin (which we prepared from whole porcine blood), and then washed with PBS. 5*10^5^ HL-60 cells were plated on each dish in normal growth media and allowed to adhere for 10 min at 37°C with 10 nM fMLP (F3506, Sigma). After, cells were washed and probed in RPMI with 2% FBS with 10 nM fMLP (F3506, Sigma) at 30°C.

Olympus BioLevers (k = 60 pN/nm) were calibrated using the thermal noise method and incubated in 2.5 mg/ml Concanavalin A (C5275, Sigma) for 1 h at RT. Before the measurements, cantilevers were rinsed in DPBS.

Cells were located by brightfield imaging, and the cantilever was positioned at any location over the cell for tether measurement. Cells were not used longer than 1 h for data acquisition.

Tethers were pulled using a Bruker Catalyst AFM controlled by custom-made LabVIEW software mounted on an inverted Zeiss fluorescent microscope. Approach velocity was set to 1 μm/s, contact force to 100 pN, contact time to 5–10 s and retraction speed to 10 μm/s. We used the signature of tether breaking in the AFM traces to identify cells that have multiple tethers by visualizing multiple steps in the force trace—these cells with multiple tethers were excluded from analysis. We titrated the productive cantilever/cell interactions to enrich for single tethers by titrating the amount of Concanavilin A used to coat the cantilever, the amount of FBS blocker in the media during tether pulling, and the force clamp time (5–10 s). After a 10 μm tether was pulled, the cantilever position was held constant until it broke. Only tethers that broke in less than 15 s were taken into account as actin polymerized inside of longer-lived tethers. The jump in force during phase II is the result of a viscoelastic response of the cell once the cantilever touches it. Negative and positive forces relate to the angle the cantilever takes, but the sign is arbitrary. By convention, contacting the cell deflects the cantilever towards positive values. Conversely, when the cantilever is pulled downwards by a membrane tether, the values are negative. Resulting force–time curves were analyzed with the Kerssemakers algorithm (Kerssemakers et al., 2006 [39]) kindly provided by Jacob Kerssemakers.

### Graphing and Statistical Analyses

Graphing and statistical analyses were performed using R, Microsoft Excel, and MATLAB.

*P*-values were calculated using *t* test after ensuring normality of data with a Shapiro-Wilk test. Otherwise, a non-parametric Mann-Whitney test was used, as it does not assume a particular data distribution.

Boxes in all box plots extend from the 25th to 75th percentiles, with a line at the median. Whiskers extend to ×1.5 IQR (interquartile range) or the max/min data points if they fall within ×1.5 IQR.

### Mathematical Modeling and Wave Pattern Analysis

All simulations and analysis steps were performed in MATLAB (MathWorks, R2014b). For a detailed description of the model, see S1 Text. All parameter values are listed in S1 Table.

All model variables were initially set to zero except for a point source of membrane-bound WAVE2. We applied periodic boundary conditions on a fixed domain in 2D for the spatial variables, polymerized actin, and membrane-bound WAVE2 complex. Stochastic simulations were performed 20 times, with starting times and times of osmotic shock or onset of competition randomized over a short initial period (2 min) (except for S6A Fig). Quantitative analysis was restricted to time-points after an initial transient or a transient after shock or onset of competition.

To quantify wave patterns of actin nucleation (see Fig 5C–5E), we developed the Wave Index (WI) as follows: At each time point, the spatial pattern (a grayscale image) is filtered by a Gaussian and threshold-segmented by a low-level intensity threshold to obtain the number of connected components in each image, which is normalized by the segmented area. This measure is based on the reasoning that images showing clear wave-like patterns have a high level of connectedness and, thus, a small number of connected components. Since the WI is sensitive to the signal-to-noise quality of the images, in the analysis of model-generated wave patterns (Figs 5E, S6, S7B and S8), we added zero-mean Gaussian noise instead of the filter, to make the WAVE2 signal-to-noise in the simulations more comparable to experimental data.

## Supporting Information

S1 Data Excel file with values used to make all plots in all figures. (XLSX)

S1 Fig Western blots to assess knockdown efficiency, pAkt S473 upon chemoattractant, transwell assays, and CD11b staining to assess cell differentiation. **(A,B)** Representative complete western blot against Rictor (A) or PLD2 (B) protein and Akt as loading control. **(C)** Median of the pAkt 473 immunofluorescence peak (readout of mTORC2 activity) before and after a 10 nM chemoattractant (normalized to Nonsense shRNA untreated cells). Mean ± SEM. Rictor and PLD2 are required for chemoattractant-induced increase in mTORC2 activation (*p* < 0.05). **(D,E)** Chemotaxis (10 nM fMLP in lower compartment) and basal migration (no chemoattractant in lower compartment) in control (Nonsense, Ns) versus Rictor (D) and PLD2 (E) shRNA cells. Mean ± SEM. Rictor and PLD2 are not required for basal migration (*p* > 0.1). (**F)** CD 11b staining to assess cell differentiation of control (Nonsense, Ns), Rictor, and PLD2 shRNA undifferentiated and differentiated cells. Rictor and PLD2 shRNA cells show no differentiation defect. N_biological replicates_: C = 6 (Rictor shRNA) and 5 (PLD2 shRNA). D,E = 6 (basal) and 7 (chemotaxis). N_cells_: C > 25,000 cells. D,E > 300,000. Statistics: Mann-Whitney test (C) and *t* test (D, E).
(TIF)

S2 Fig Adhesion is not the main generator of membrane tension in neutrophils. **(A)** Titration of surface density of fluorescently labeled fibronectin. Mean. **(B)** Cell adhesion for cells plated on different fibronectin densities. Mean ± SEM. **(C)** Migration speed for stimulated cells plated on different fibronectin densities. **(D)** Static tether force for stimulated cells plated on different fibronectin densities. No change in measure membrane tension can be found across this 10-fold range of fibronectin density (*p* > 0.1). N_biological replicates_: B,C = 2, D = 3. N_cells_: B = 328 (0%BSA), 229 (5%BSA), C = 9 (0%BSA), 9 (5%BSA), D = 17 (0%BSA), 21 (5%BSA). N_tethers_: D = 38 (0%BSA), 45 (5%BSA). Statistics: *t* test (B,C) and Mann-Whitney test (D). Boxes in all box plots (B,C,D) extend from the 25th to 75th percentiles, with a line at the median. Whiskers extend to ×1.5 IQR (interquartile range) or the max/min data points if they fall within ×1.5 IQR.
(TIF)

S3 Fig PLD2 inhibition by VU0285655-1 recapitulates the higher membrane tension phenotype of PLD2 shRNA. **(A)** Static tether force for stimulated DMSO-treated control and VU0285655-1 treated cells. PLD2 inhibited cells have significantly increased membrane tension (*p* < 0.01). N_biological replicates_ = 3. N_cells_ = 19 (DMSO control), 20 (VU0285655-1). N_tethers_: D = 44 (DMSO control), 67 (VU0285655-1). Statistics: Mann-Whitney test and *t* test. Boxes in all box plots (B,C,D) extend from the 25th to 75th percentiles, with a line at the median. Whiskers extend to ×1.5 IQR (interquartile range) or the max/min data points if they fall within ×1.5 IQR.
(TIF)

S4 Fig Complete time series of Hem-1 GFP loss upon 70 mOsm hypo-osmotic shock. Hem1-GFP detachment from the membrane upon 70 mOsm hypo-osmotic shock in example control (Nonsense, Ns) (A), Rictor (B), and PLD2 (C) shRNA cells. Scalebar = 10 μm. Time in seconds before and after osmotic shock.
(TIF)

S5 Fig Modeling actin wave nucleation with global feedback. (A) Model scheme: We simulate actin wave generation in a small, representative portion of a leading edge. The average level of polymerized actin in that region is used to estimate the cellular membrane tension, which gives rise to elevated mTORC2 activation (see S1 Text for details of the model). (B) Simulation of Model I. Coherent wave patterns can be observed early in the simulation [40]. (C) Linear regression of membrane tension versus polymerized actin, values obtained from Figs 1–3. For model calibration (parameters *α* and β in S1 Table), phalloidin fluorescence was converted to fraction of actin polymerization by assuming that in wt cells 50% of the actin is polymerized (see S1 Text). (D) Dependence of the direct feedback factor *f*_*A*_*(t)* and the indirect feedback factor *f*_*H*_*(x(T))* on membrane tension. Here we used the steady-state value of Eq 6 to calculate x(T), as described in Section II. Mean ± SD of 20 stochastic simulations. (E) Median of phalloidin staining before and 3 and 10 min after fMLP stimulation. LatB and CK666 treated cells have lower amounts of polymerized actin than DMSO-treated control cells (*p* < 0.05). Mean ± SEM. (F) Static tether force for stimulated DMSO-treated control, 50 nM LatB, and 100 μM CK666 treated cells. LatB and CK666 treated cells have significantly lower membrane tension (*p* < 0.01). **(G,H)** Nonlinear regression of membrane tension versus mTORC2 activity as assessed by pAkt S473 staining using the formula mTORC2 = v_max * tension^h/(K^h + tension^h) + 1. (**G**) Data and best curve (h = 7, dashed line). (**H**) Standard error from curve fitting for a range of fixed Hill coefficients h. **(I)** Parameter estimation: The mTORC2 disassociation constant d_x_ was obtained by exponential fitting as described in Section II. N_biological replicates_: E = 6 (CK666) and 5 (LatB), F = 3. N_cells_: E >10,000/data point, F = 19 (DMSO control), 11 (CK666) and 11 (LatB). N_tethers_: F = 44 (DMSO control), 26 (CK666), and 17 (LatB). Statistics: t test (E,F) and Mann-Whitney test (F). Boxes in all box plots (F) extend from the 25th to 75th percentiles, with a line at the median. Whiskers extend to ×1.5 IQR (interquartile range) or the max/min data points if they fall within ×1.5 IQR.
(TIF)

S6 Fig Response of models to hypo-osmotic shock and feedback analysis. **(A)** Full model kinetics of simulations in Fig 5B. The dynamics of the system can be broadly grouped as: the initial transient (“tr. 1”), the time before osmotic shock (“Before”), a short transient after osmotic shock (“tr. 2”), and the time after osmotic shock (“After”). Wave patterns shown in Fig 5C are snapshots at the end of the “before” and “after” osmotic shock dynamics. Inset contains curves with large-scale changes in the *y*-axis. (**B)** Feedback analysis. Variation of either the direct or the indirect feedback strength (parameters *k*_*i*,*A*_ and *k*_*i*,*H*_, respectively), in which one of those parameters is held constant at the standard value k_i_ = 20. All other parameter values are in S1 Table. Labels refer to the model topologies defined in Fig 5A and indicate the direct and indirect feedback strength in the respective model. “After” denotes after osmotic shock.
(TIF)

S7 Fig Sensitivity analysis of mathematical model. (**A**) Simulation of osmotic shock experiments (see Figs 5B and S6A) with a range of Hill coefficients n (see Eq 7 in S1 Text). (**B,C**) Model simulation up to stationary state (see Fig 5B and 5E) with 50% reduced or increased values of parameters that are not set by experimental data in this work (cf. S1 Table). (**B**) Stationary values for WAVE2 on membrane and Wave Index, (**C**) ratio of stationary values before/after osmotic shock (see Fig 5E).
(TIF)

S8 Fig Simulated competition between two nucleating regions that share membrane tension. Simulated response of two spatially separate sites of actin assembly that are linked only via membrane tension. Wave Index is shown for one protrusion growing in isolation (region 1 alone, black) or following equivalent activation of polymerization in a second region (activity of region 1 followed by activation of region 2, grey) as in Fig 7. See S6 Movie. Mean ± SD of 20 stochastic simulations. Statistics: *t* test.
(TIF)

S1 Movie Reducing the media osmolarity from 280 to 140 and 70 mOsm by adding water. 140 mOsm does not lead to a loss of polarity, but 70 mOsm does. Scale bar = 10 μm. Time in minutes:seconds.
(MP4)

S2 Movie WAVE2 complex (Hem 1-GFP) dynamics in control (Nonsense shRNA) cells. Scale bar = 10 μm. Time in minutes:seconds.
(MP4)

S3 Movie WAVE2 complex (Hem 1-GFP) dynamics in Rictor shRNA cells. Scale bar = 10 μm. Time in minutes:seconds.
(MP4)

S4 Movie WAVE2 complex (Hem 1-GFP) dynamics in PLD2 shRNA cells. Scale bar = 10 μm. Time in minutes:seconds.
(MP4)

S5 Movie Simulation of the behavior of the WAVE2 complex in the different models. Membrane tension is increased by 80 μN/m to simulate “osmotic shock” after 20 min of simulation time.
(MP4)

S6 Movie Simulation of the behavior of the WAVE2 complex during competition between two nucleating regions that share membrane tension. (MP4)

S1 Table Parameters used in the model and source. (DOCX)

S1 Text Supplemental text file with detailed description of the model. (DOCX)

## Abbreviations

AFM: atomic force microscope
DMSO: dimethyl sulfoxide
fMLP: formyl-Met-Leu-Phe
mTORC2: mammalian target of rapamycin complex 2
PDMS: polydimethylsiloxane
PLD2: phospholipase D2
shRNA: small hairpin RNA
TIRF: total internal reflection fluorescence
WI: Wave Index
