# Supplementary figures and images for: Membrane Tension Acts Through PLD2 and mTORC2 to Limit Actin Network Assembly During Neutrophil Migration
Source: PLoS Biol. 2016 Jun 9;14(6):e1002474. doi: 10.1371/journal.pbio.1002474 (PMC4900667; doi:10.1371/journal.pbio.1002474)

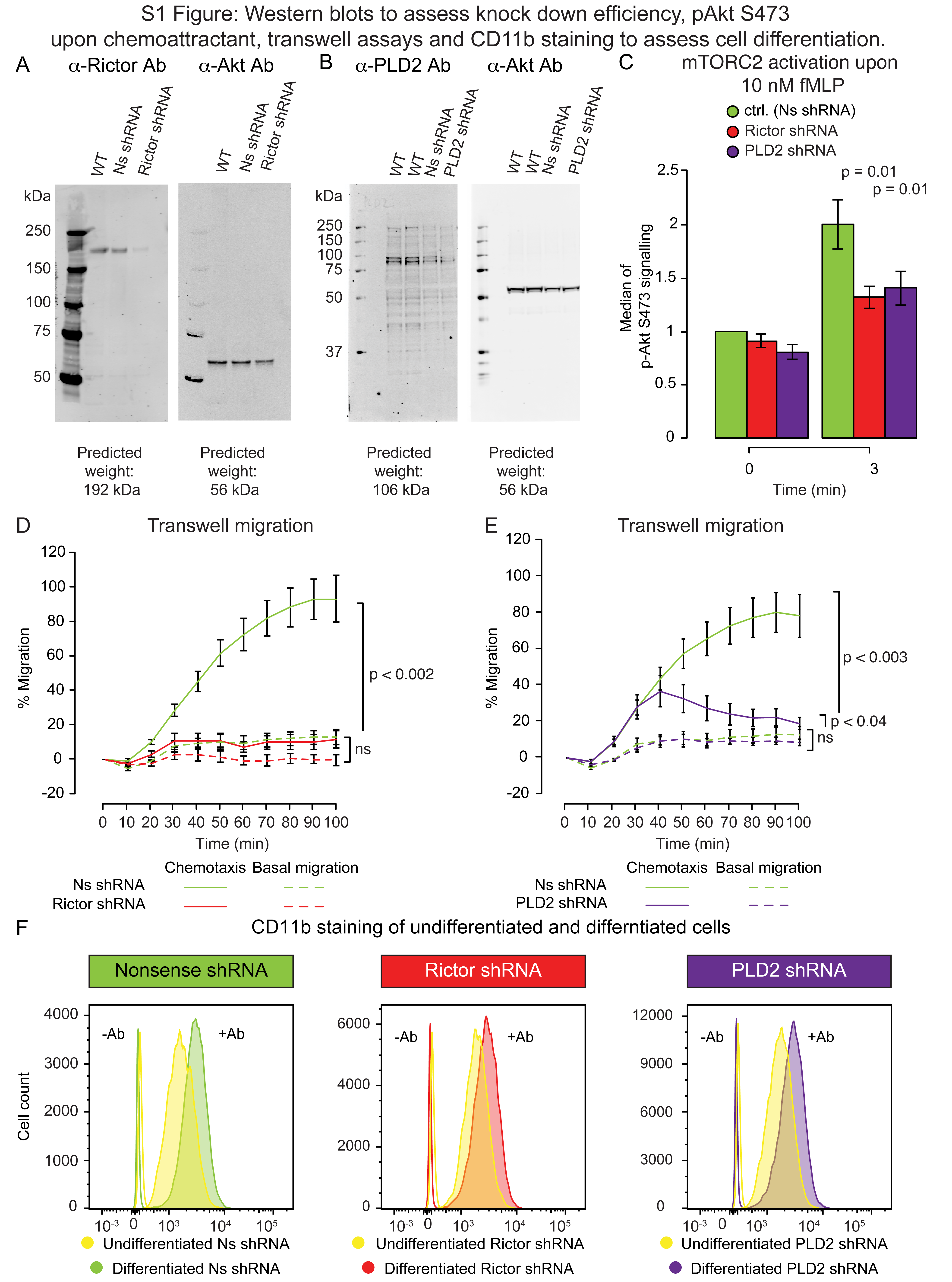

Supplement: S1 Fig — (A,B) Representative complete western blot against Rictor (A) or PLD2 (B) protein and Akt as loading control. (C) Median of the pAkt 473 immunofluorescence peak (readout of mTORC2 activity) before and after a 10 nM chemoattractant (normalized to Nonsense shRNA untreated cells). Mean ± SEM. Rictor and PLD2 are required for chemoattractant-induced increase in mTORC2 activation (p < 0.05). (D,E) Chemotaxis (10 nM fMLP in lower compartment) and basal migration (no chemoattractant in lower compartment) in control (Nonsense, Ns) versus Rictor (D) and PLD2 (E) shRNA cells. Mean ± SEM. Rictor and PLD2 are not required for basal migration (p > 0.1). (F) CD 11b staining to assess cell differentiation of control (Nonsense, Ns), Rictor, and PLD2 shRNA undifferentiated and differentiated cells. Rictor and PLD2 shRNA cells show no differentiation defect. Nbiological replicates: C = 6 (Rictor shRNA) and 5 (PLD2 shRNA). D,E = 6 (basal) and 7 (chemotaxis). Ncells: C > 25,000 cells. D,E > 300,000. Statistics: Mann-Whitney test (C) and t test (D, E). (TIF) [file pbio.1002474.s002.tif]

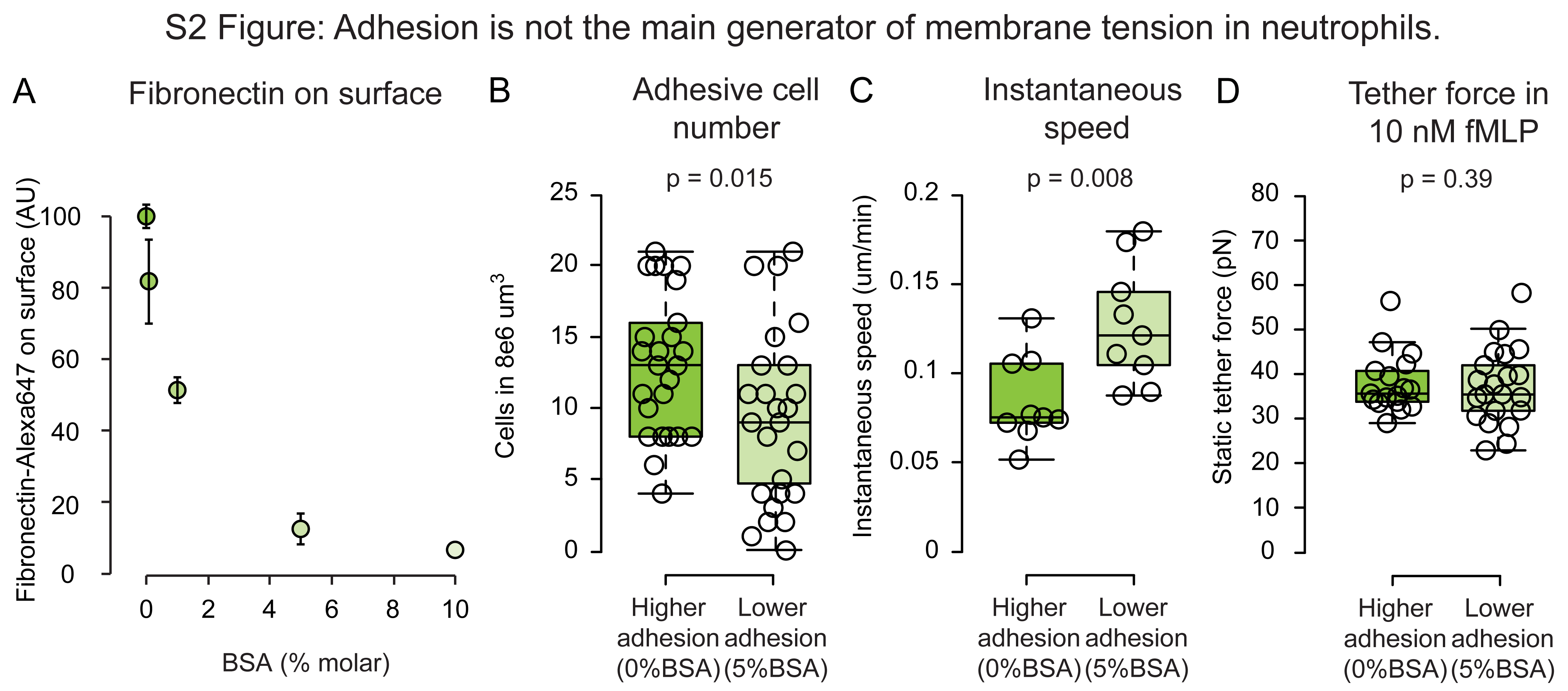

Supplement: S2 Fig — (A) Titration of surface density of fluorescently labeled fibronectin. Mean. (B) Cell adhesion for cells plated on different fibronectin densities. Mean ± SEM. (C) Migration speed for stimulated cells plated on different fibronectin densities. (D) Static tether force for stimulated cells plated on different fibronectin densities. No change in measure membrane tension can be found across this 10-fold range of fibronectin density (p > 0.1). Nbiological replicates: B,C = 2, D = 3. Ncells: B = 328 (0%BSA), 229 (5%BSA), C = 9 (0%BSA), 9 (5%BSA), D = 17 (0%BSA), 21 (5%BSA). Ntethers: D = 38 (0%BSA), 45 (5%BSA). Statistics: t test (B,C) and Mann-Whitney test (D). Boxes in all box plots (B,C,D) extend from the 25th to 75th percentiles, with a line at the median. Whiskers extend to ×1.5 IQR (interquartile range) or the max/min data points if they fall within ×1.5 IQR. (TIF) [file pbio.1002474.s003.tif]

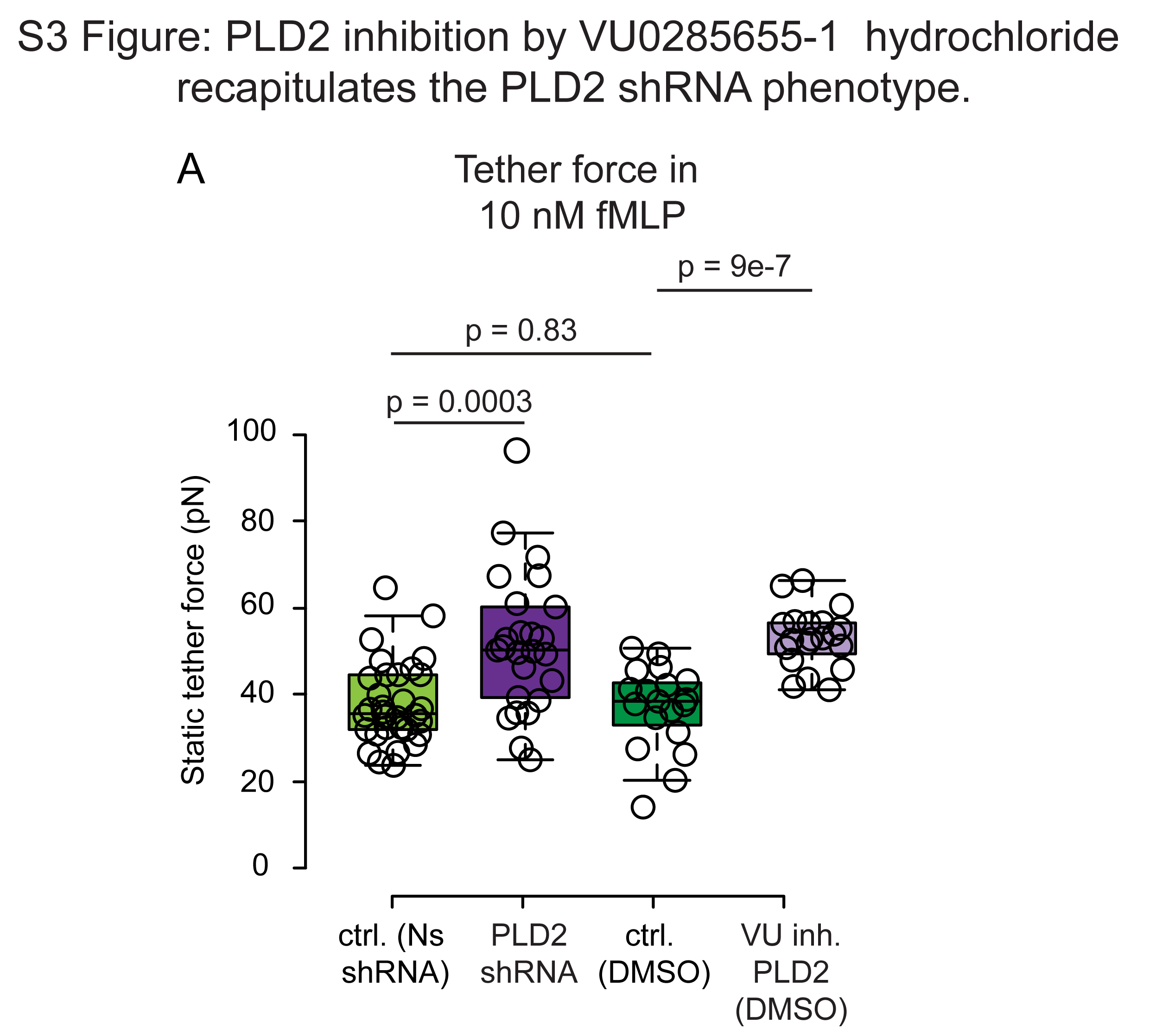

Supplement: S3 Fig — (A) Static tether force for stimulated DMSO-treated control and VU0285655-1 treated cells. PLD2 inhibited cells have significantly increased membrane tension (p < 0.01). Nbiological replicates = 3. Ncells = 19 (DMSO control), 20 (VU0285655-1). Ntethers: D = 44 (DMSO control), 67 (VU0285655-1). Statistics: Mann-Whitney test and t test. Boxes in all box plots (B,C,D) extend from the 25th to 75th percentiles, with a line at the median. Whiskers extend to ×1.5 IQR (interquartile range) or the max/min data points if they fall within ×1.5 IQR. (TIF) [file pbio.1002474.s004.tif]

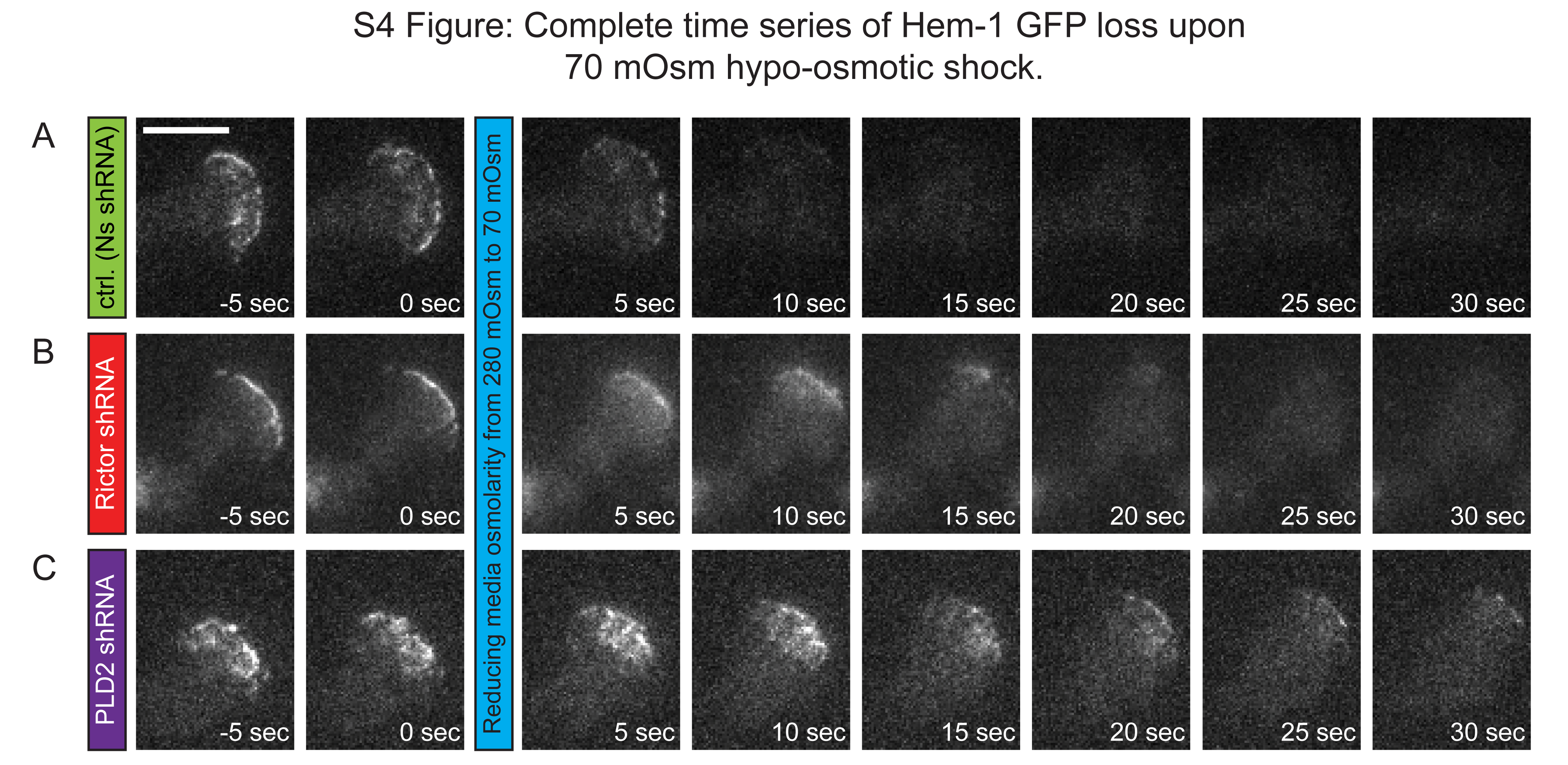

Supplement: S4 Fig — Hem1-GFP detachment from the membrane upon 70 mOsm hypo-osmotic shock in example control (Nonsense, Ns) (A), Rictor (B), and PLD2 (C) shRNA cells. Scalebar = 10 μm. Time in seconds before and after osmotic shock. (TIF) [file pbio.1002474.s005.tif]

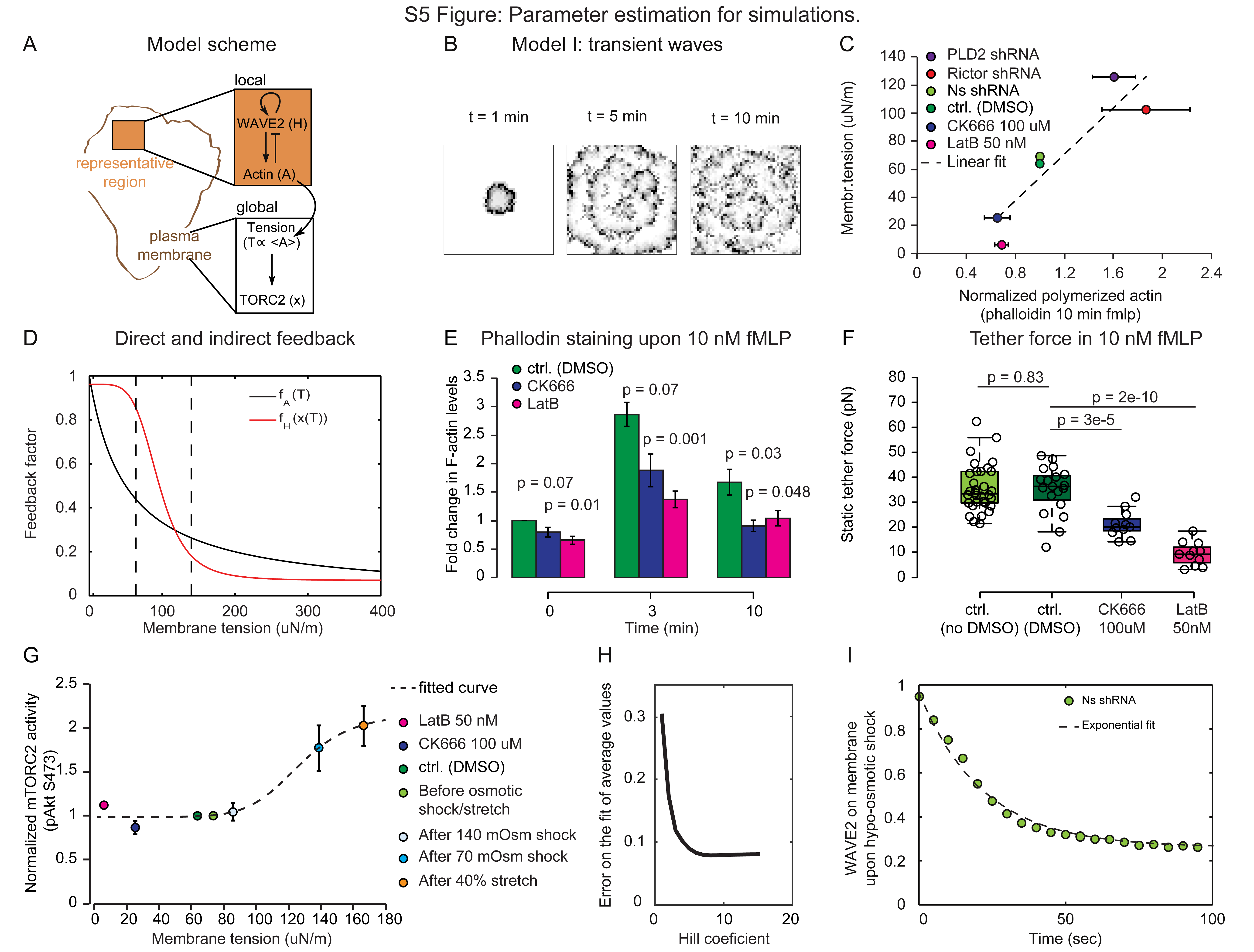

Supplement: S5 Fig — (A) Model scheme: We simulate actin wave generation in a small, representative portion of a leading edge. The average level of polymerized actin in that region is used to estimate the cellular membrane tension, which gives rise to elevated mTORC2 activation (see S1 Text for details of the model). (B) Simulation of Model I. Coherent wave patterns can be observed early in the simulation [40]. (C) Linear regression of membrane tension versus polymerized actin, values obtained from Figs 1–3. For model calibration (parameters α and β in S1 Table), phalloidin fluorescence was converted to fraction of actin polymerization by assuming that in wt cells 50% of the actin is polymerized (see S1 Text). (D) Dependence of the direct feedback factor fA(t) and the indirect feedback factor fH(x(T)) on membrane tension. Here we used the steady-state value of Eq 6 to calculate x(T), as described in Section II. Mean ± SD of 20 stochastic simulations. (E) Median of phalloidin staining before and 3 and 10 min after fMLP stimulation. LatB and CK666 treated cells have lower amounts of polymerized actin than DMSO-treated control cells (p < 0.05). Mean ± SEM. (F) Static tether force for stimulated DMSO-treated control, 50 nM LatB, and 100 μM CK666 treated cells. LatB and CK666 treated cells have significantly lower membrane tension (p < 0.01). (G,H) Nonlinear regression of membrane tension versus mTORC2 activity as assessed by pAkt S473 staining using the formula mTORC2 = v_max * tension^h/(K^h + tension^h) + 1. (G) Data and best curve (h = 7, dashed line). (H) Standard error from curve fitting for a range of fixed Hill coefficients h. (I) Parameter estimation: The mTORC2 disassociation constant dx was obtained by exponential fitting as described in Section II. Nbiological replicates: E = 6 (CK666) and 5 (LatB), F = 3. Ncells: E >10,000/data point, F = 19 (DMSO control), 11 (CK666) and 11 (LatB). Ntethers: F = 44 (DMSO control), 26 (CK666), and 17 (LatB). Statistics: t test (E,F) and Mann-Whi [file pbio.1002474.s006.tif]

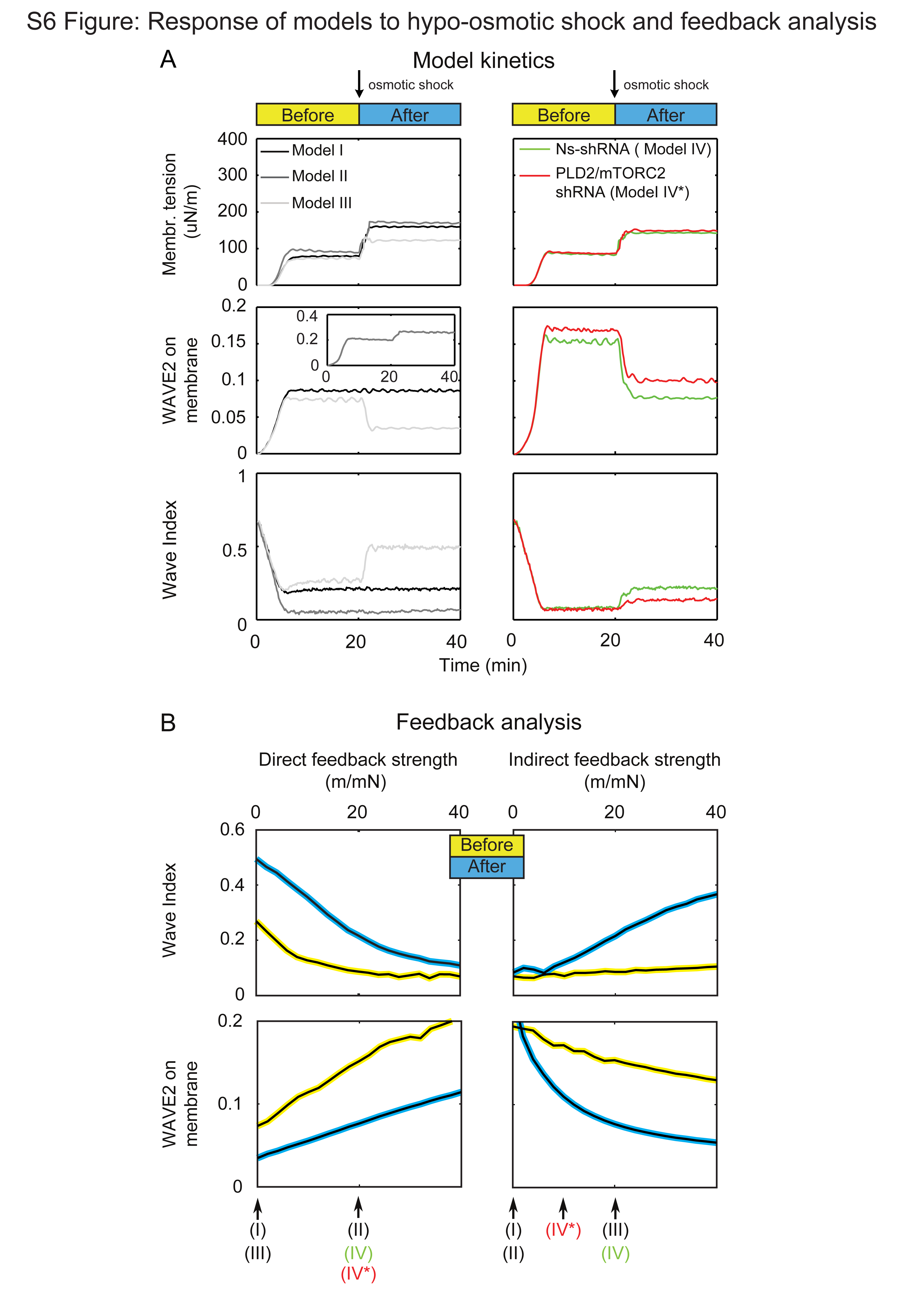

Supplement: S6 Fig — (A) Full model kinetics of simulations in Fig 5B. The dynamics of the system can be broadly grouped as: the initial transient (“tr. 1”), the time before osmotic shock (“Before”), a short transient after osmotic shock (“tr. 2”), and the time after osmotic shock (“After”). Wave patterns shown in Fig 5C are snapshots at the end of the “before” and “after” osmotic shock dynamics. Inset contains curves with large-scale changes in the y-axis. (B) Feedback analysis. Variation of either the direct or the indirect feedback strength (parameters ki,A and ki,H, respectively), in which one of those parameters is held constant at the standard value ki = 20. All other parameter values are in S1 Table. Labels refer to the model topologies defined in Fig 5A and indicate the direct and indirect feedback strength in the respective model. “After” denotes after osmotic shock. (TIF) [file pbio.1002474.s007.tif]

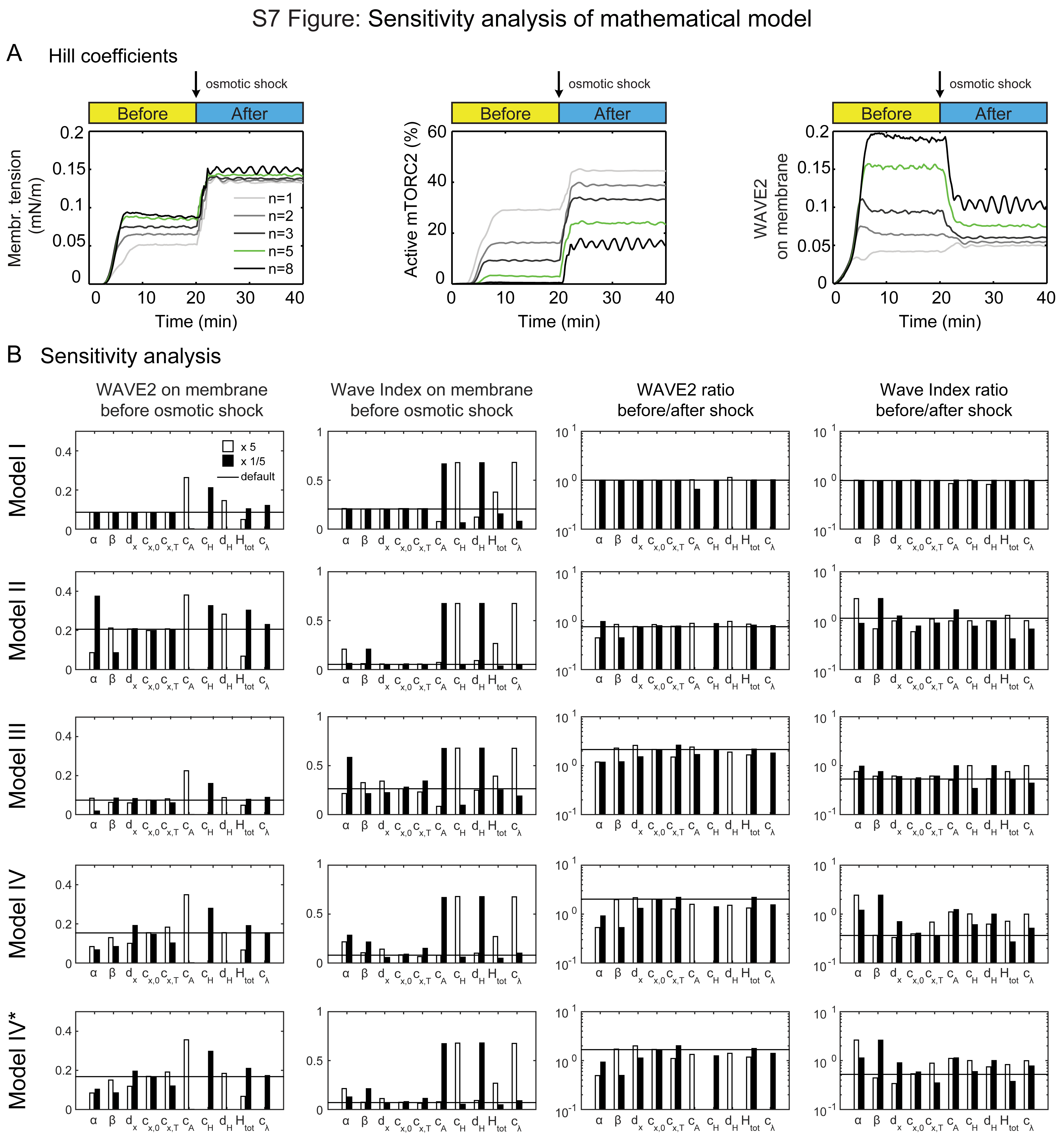

Supplement: S7 Fig — (A) Simulation of osmotic shock experiments (see Figs 5B and S6A) with a range of Hill coefficients n (see Eq 7 in S1 Text). (B,C) Model simulation up to stationary state (see Fig 5B and 5E) with 50% reduced or increased values of parameters that are not set by experimental data in this work (cf. S1 Table). (B) Stationary values for WAVE2 on membrane and Wave Index, (C) ratio of stationary values before/after osmotic shock (see Fig 5E). (TIF) [file pbio.1002474.s008.tif]

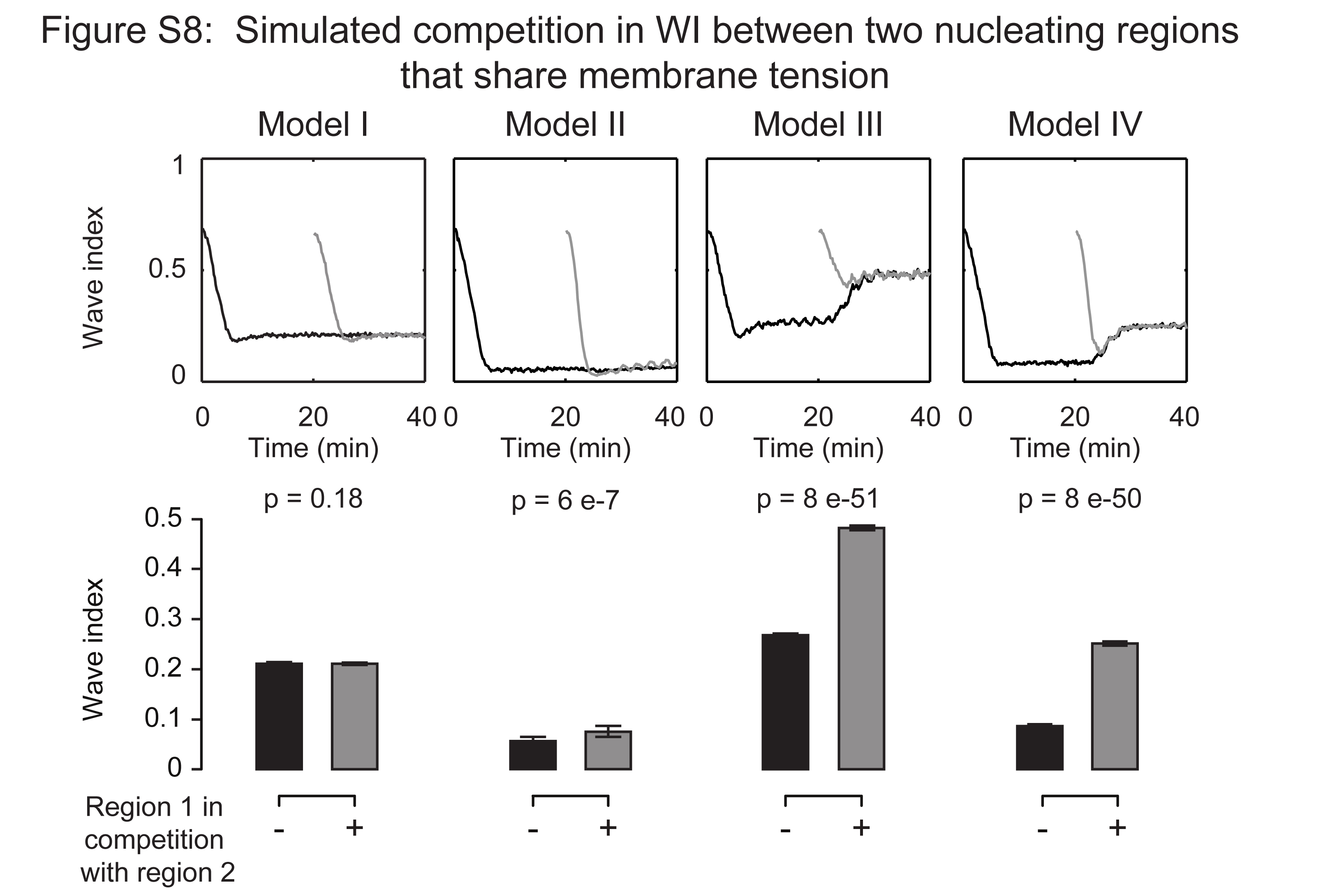

Supplement: S8 Fig — Simulated response of two spatially separate sites of actin assembly that are linked only via membrane tension. Wave Index is shown for one protrusion growing in isolation (region 1 alone, black) or following equivalent activation of polymerization in a second region (activity of region 1 followed by activation of region 2, grey) as in Fig 7. See S6 Movie. Mean ± SD of 20 stochastic simulations. Statistics: t test. (TIF) [file pbio.1002474.s009.tif]
