# Supplementary material for: Membrane Tension Acts Through PLD2 and mTORC2 to Limit Actin Network Assembly During Neutrophil Migration
Source: PLoS Biol. 2016 Jun 9;14(6):e1002474. doi: 10.1371/journal.pbio.1002474 (PMC4900667; doi:10.1371/journal.pbio.1002474)
Supplement: S1 Table — (DOCX) [file pbio.1002474.s016.docx]

S1 Table: Parameters used in the model and source

| **Data used for model calibration** | **Symbol** | **Value** | **Unit** | **Data source** |
| --- | --- | --- | --- | --- |
| Membrane tension of Ns shRNA cells | T_Ns | 0.069 | mN/m | Fig 2F ^(a)^ |
| Membrane tension of Rictor shRNA cells | T_Rictor | 0.103 | mN/m | Fig 2F ^(a)^ |
| Membrane tension of PLD2 shRNA cells | T_PLD2 | 0.126 | mN/m | Fig 3F ^(a)^ |
| Actin ratio (PLD2 shRNA/Ns shRNA cells) | r_actin_PLD2 | 1.553 |  | Fig 3E |
| Actin ratio (Rictor shRNA/Ns shRNA cells) | r_actin_Rictor | 1.780 |  | Fig 2E |
| Membrane tension before 70 mOsm hypo-osmotic shock | T_before | 0.064 | mN/m | Fig 1F ^(a)^ |
| Membrane tension after 70 mOsm hypo-osmotic shock | T_after | 0.139 | mN/m | Fig 1F ^(a)^ |
| WAVE2 complex dissociation upon 70 mOsm hypo-osmotic shock | k_TORC_deg | 2.76 | 1/min | Fig 4D and S3D ^(a)^ |
|  | | | | |
| **Model parameters** | **Symbol** | **Value** | **Unit** | **Data source** |
| Plasma membrane tension constant | α | 0.18 | mN/m | Fig S3D ^(a)^ |
| Plasma membrane tension offset | β | 0.03 | mN/m |  |
| Base-level mTORC2 complex activation rate | c_{x,0} | 0.006 | 1/min | unknown |
| Membrane tension-induced mTORC2 complex activation rate | c_{x,T} | 6 | 1/min | unknown |
| mTORC2 complex inactivation rate | d_x | 3 | 1/min | Fig 4D and S3D ^(a)^ |
| Hill coefficient for mTORC2 complex activation | n | 5 |  | unknown |
| Half-saturation constant for mTORC2 complex activation | K_TORC | 0.2 |  | unknown |
| Actin polymerization rate constant | c_A | 1 | 1/(s molecules) | Weiner et al. 2007 |
| WAVE2 complex membrane binding rate | c_H | 1 | 1/(s molecules) | Weiner et al. 2007 |
| WAVE2 complex membrane disassociation rate | d_H | 0.36 | 1/min | Weiner et al. 2007 |
| Total amount of WAVE2 complex molecules | H_tot | 1500 | molecules | Weiner et al. 2007 |
| WAVE2 complex nucleation probability | c_λ | 1 | 1/s | Weiner et al. 2007 |
| Tension increase by osmotic shock | ΔT | 0.08 | mN/m | Difference T_after and T_before |

^(a)^: see Section II for details
