# Supplementary material for: Membrane Tension Acts Through PLD2 and mTORC2 to Limit Actin Network Assembly During Neutrophil Migration
Source: PLoS Biol. 2016 Jun 9;14(6):e1002474. doi: 10.1371/journal.pbio.1002474 (PMC4900667; doi:10.1371/journal.pbio.1002474)
Supplement: S1 Text — (DOCX) [file pbio.1002474.s017.docx]

Supplemental information:

Membrane tension acts through PLD2 and mTORC2 to limit actin network assembly during neutrophil migration

Alba Diz-Muñoz, Kevin Thurley, Sana Chintamen, Steven J. Altschuler, Lani F. Wu, Daniel A. Fletcher, and Orion D. Weiner

SECTION I: MODEL DESCRIPTION

Since our goal was to study consequences of the newly discovered indirect feedback loop (from membrane tension through PLD2 and mTORC2 to WAVE2 complex activation) in a qualitative manner we started from the generic actin wave generator developed in Weiner *et al.* [1] rather than describing all reaction steps in mechanistic detail. We adjusted the model in order to specifically describe a representative portion of the membrane. Further, we extended the model by adding global (cell-wide) feedback inhibition from membrane tension on WAVE2 complex membrane binding and actin polymerization, either directly or mediated by the PLD2-mTORC2 pathway described in this study. To constrain the model, we estimated parameters describing the additional global feedback mechanisms from the data in this study wherever possible (see Section II and **S1 Table**).

*Model geometry and equations:*

The model in Ref. [1] describes a stochastic wave generator acting by a combination of short-range auto-activation and local inhibition. We modeled a portion of the cell membrane as a fixed-size rectangular domain (**S5A Fig**). We chose to make use of periodic boundary conditions as they enable equalization of outward and inward fluxes of molecular components (which is reasonable for a small region inside a larger field with similar properties, here the whole leading edge) and avoid disruption of observed spatial organization near the boundary. In each region, we considered the average amounts of membrane-bound actin and WAVE2 complex as core variables of the model.

The resulting model (model I in **Fig 5**) contains only local feedback and serves as a starting point for our analysis. It can be written as follows:

| $\frac{\partial A}{\partial t}=c_{A}H\left( 1-A \right)$ $\frac{\partial H}{\partial t}=\left[ c_{H}\left( 1-A \right)H_{c}\left( H*G_{SA} \right)-d_{H}AH \right]\delta_{nuc,1}$  $Prob\left( nuc=1\vert nuc=0,t+dt\vert t \right) \sim Poiss\left( \lambda dt \right)$ $Prob\left( nuc=0\vert nuc=1,t+dt\vert t \right)=\left\{ \begin{aligned} 1, H<H_{thresh} \\ 0, otherwise \end{aligned} \right.$ |  | \| (1) \| \| --- \| \| (2) \| \| (3) \| \| (4) \| |
| --- | --- | --- | --- | --- | --- | --- |
| $\lambda(p,t)= c_{\lambda}H_{c}\left( H*G_{SA} \right)$ |  | (5) |

Here, for membrane location grid point *p* and time *t*: *A(p,t)* is the fraction of polymerized actin, *H(p,t)* is the amount of membrane bound WAVE2 complex (in molecules per grid point; note *H* was used to represent Hem1 in the original model), and $H_{c}(t)=1-(\sum_{p} H(p,t))/H_{tot}$ (*H_tot_* the total number of bound and unbound WAVE2 complex molecules) is the fraction of cytosolic WAVE2 complex. *δ_i,j_* is the Kronecker Symbol, * denotes convolution, and *G_SA_* is a short-range activator kernel (Gauss kernel with radius = standard deviation = 1 pixel). *Prob(.)*~*Poiss(y)* means that random events occur following a Poisson distribution with parameter *y*. This means that the WAVE2 complex can only bind to the membrane after an initial nucleation process is completed, which is described by a Poisson process with intensity $\lambda(p,t)$. Nucleated parts of the simulated region are initialized by 0.1 molecules per grid point of the WAVE2 complex. When the WAVE2 complex falls below a threshold value *H_thresh_* = 0.01 molecules per grid point (due to local inhibition by polymerized actin), it is assumed that all WAVE2 complexes fall off until a new nucleus is formed at this part of the membrane.

*Model Extension by global feedback*

To analyze global feedback, we calculated membrane tension, *T*, and the fraction of active mTORC2 complex, *x*, by the following equations:

| $T= \alpha\left\langle A \right\rangle-\beta$ $\frac{dx}{dt}={(c}_{x,0}+c_{x,T}\frac{T^{n}}{K^{n}+T^{n}})\left( 1-x \right)-d_{x}x$ | \| (6) \| \| --- \| \| (7) \| |
| --- | --- | --- | --- |

where *<A>* is the cell-averaged fraction of polymerized actin (parameters are described in Section II and Table S1), and *T* is set to 0 for $\left\langle A \right\rangle<\beta/\alpha$. Membrane tension and active mTORC2 complex feed back into actin polymerization and WAVE2 complex membrane binding by means of two inhibitory factors *f_A_(T)* and *f_H_(x)*, in the equations for *A,H* (see below for their specific form):

| $\frac{\partial A}{\partial t}=c_{A}H\left( 1-A \right)f_{A}\left( T \right)$ $\frac{\partial H}{\partial t}=\left[ c_{H}\left( 1-A \right)H_{C}\left( H*G_{SA} \right)f_{H}\left( x \right)-d_{H}AH \right]\delta_{nuc,1}$ | \| (8) \| \| --- \| \| (9) \| |
| --- | --- | --- | --- |

This model extension is based on the following considerations:

- Upon an increase in actin polymerization (e.g. induced by a chemotactic cue like fMLP), the cell’s membrane tension increases by a value proportional to the cell-averaged level of polymerized actin *<A>* (**S5C Fig**; see also [2]). In simulations, a Hill coefficient n=5 was used for membrane tension-associated mTORC2 complex activation (larger values for n did not qualitatively change the behavior of the model, c.f. **S7A Fig** right panel).

- Upon an increase of membrane tension over a certain threshold (following stimulation, sufficiently strong hypo-osmotic shock or cell stretch), we observe a non-linear increase in mTORC2 complex activity (**Fig S5G**).

*Detailed description of models I-IV*

In all models studied here, the inhibitory factors *f_A_(T)* and *f_H_(x)* in Equations 8-9 take the form $f_{A}\left( T \right)=1/(1+k_{A}T)$, $f_{H}\left( x \right)=1/(1+k_{H}x)$. The inhibitory constant *k_A_* equals 0 in models I, III, and the constant *k_H_* equals zero in models I, II. Otherwise the constants take their standard values *k_A_*=20 m/mN and *k_H_=20,* except for model IV* where the indirect feedback strength is reduced to *k_H_=12* to reflect PLD2-mTORC2 knockdown (**Fig S6B**).

In **Fig S5D** we illustrate the effect from this type of feedback on equations 8-9. In the absence of feedback (*k_A/H_=0*), the “feedback factor” *f_A/H_* is 1. The “direct feedback” *f_A(T)_* induces a hyperbolic decay with membrane tension. The “indirect feedback” depends on tension via PLD2-mTORC2, *f_H_(x) =f_H_(x(T)).* At steady state (from **Eq. 7**), *x(T) =a(T)/(a(T)+d_x_)* where ${a\left( T \right)=c}_{x,0}+c_{x,T}\frac{T^{n}}{K^{n}+T^{n}}$. Therefore, f_H_(x(T)) has the form of a sigmoidal (or switch-like) decay with constant levels at low and high values of membrane tension.

*Cell morphology during competition*

We used a stretched-cell morphology to simulate competition, because in this geometry, competition only occurs through membrane tension, not exchange of soluble components.  Furthermore, we know that this morphology is compatible with competition in real cells [3].

SECTION II: PARAMETER IDENTIFICATION

1) Membrane tension

We estimated the membrane tension using the following formula:

| $T=\frac{F_{0}^{2}}{8B\pi^{2}}$ | (10) |
| --- | --- |

where *F_0_* is the tether force measured by force spectroscopy using and atomic force microscope (AFM), and *B* is the bending rigidity of the membrane, which we assume is invariant between the different cell lines tested (= 2.7 10^-19^ Nm [4]).

We found that the tether force in control (Ns shRNA) cells is smaller than in Rictor and PLD2 shRNA cells (**Fig 2F** and **3F**). The smaller membrane tension in Ns shRNA is due to reduced actin polymerization, which we observed by phalloidin staining (**Fig 2E**, **3E and S5C, E and F**). We used these data to fit **Eq. 6** to the value pairs of membrane tension and polymerized actin **(S5C Fig**).

2) mTORC2 complex inactivation

In **Fig 4D,** we measured the dynamical response of membrane-bound WAVE2 complex following osmotic shock. Empirically, we found that this data is well explained by a single exponential decay of the form (**S5I Fig**):

| Y$\left( t \right)= a+\left( 1-a \right)e^{-bt}$(11) |  |
| --- | --- |

Assuming that this decay in the amount of membrane bound WAVE2 complex is mainly a consequence of membrane tension-induced mTORC2 complex activation and subsequent global feedback (**Eq. 9**). We obtain the time constant of mTORC2 dynamics from this fit; namely we set $d_{x}=b$ (see **S1 Table**). Note that this value is only used as a rule-of-thumb estimate for the time-scale of the simulations; our analysis and conclusions are independent of the dynamics and focus on qualitative changes in time-averaged WAVE2 levels and wave patterning after transient responses to perturbations (see main text).

REFERENCES

1. Weiner OD, Marganski WA, Wu LF, Altschuler SJ, Kirschner MW (2007) An actin-based wave generator organizes cell motility. Plos Biol 5: e221. doi:10.1371/journal.pbio.0050221.

2. Lieber AD, Yehudai-Resheff S, Barnhart EL, Theriot JA, Keren K (2013) Membrane tension in rapidly moving cells is determined by cytoskeletal forces. Curr Biol 23: 1409–1417. doi:10.1016/j.cub.2013.05.063.

3. Houk AR, Jilkine A, Mejean CO, Boltyanskiy R, Dufresne ER, et al. (2012) Membrane tension maintains cell polarity by confining signals to the leading edge during neutrophil migration. Cell 148: 175–188. doi:10.1016/j.cell.2011.10.050.

4. Hochmuth FM, Shao JY, Dai J, Sheetz MP (1996) Deformation and flow of membrane into tethers extracted from neuronal growth cones. Biophys J 70: 358–369. doi:10.1016/S0006-3495(96)79577-2.
